# Heterojunction‐Engineered Mass Spectrometry Platform for Deciphering Serum Metabolic Fingerprints in Diagnosis of Respiratory Diseases

**DOI:** 10.1002/advs.75597

**Published:** 2026-05-08

**Authors:** Junyu Chen, Chaoqi Wang, Xi Yu, Yuming Jiang, Yijiao Qu, Huihui Fu, Yuyin Bu, Xiaoyong Zhang, Zongxiu Nie

**Affiliations:** ^1^ Jiangxi Province Key Laboratory of Immunology and Inflammation Jiangxi Provincial Clinical Research Center For Laboratory Medicine Department of Clinical Laboratory, The Second Affiliated Hospital, Jiangxi Medical College Nanchang University Nanchang China; ^2^ Beijing National Laboratory for Molecular Sciences Key Laboratory of Analytical Chemistry For Living Biosystems Institute of Chemistry Chinese Academy of Sciences Beijing China; ^3^ Department of Computational Biomedicine Smidt Heart Institute and Advanced Clinical Biosystems Research Institute Cedars Sinai Medical Center Los Angeles California USA; ^4^ Department of Chemistry Nanchang University Nanchang China; ^5^ State Key Laboratory of Tropic Ocean Engineering Materials and Materials Evaluation School of Marine Science and Engineering Hainan University Haikou China

**Keywords:** disease diagnosis, laser desorption/ionization mass spectrometry, metabolic profiling, metal–organic framework hybrids, respiratory diseases

## Abstract

Respiratory diseases, including bronchial asthma (BA), chronic obstructive pulmonary disease (COPD), interstitial lung disease (ILD), and lung cancer (LCa), pose a major global health challenge due to overlapping symptoms that frequently delay accurate diagnosis. Although metabolomics‐based molecular phenotyping offers a promising path forward, its application in clinical treatment is limited by the use of conventional analytical techniques. Herein, MOF‐derived metal oxide/TiO_2_ heterojunctions (including ZnTi, FeTi, CrTi, CuTi, and CoTi) are synthesized and evaluated as a nanomatrix for high‐throughput laser desorption/ionization mass spectrometry (LDI‐MS). CoTi is found to exhibit enhanced laser absorption, suppress charge recombination, improve photothermal desorption, and have a high tolerance to complex biofluids. This platform enables high‐quality serum metabolic fingerprints to be acquired from 776 clinical samples, accurately discriminates BA, COPD, ILD, and LCa from healthy controls, and precisely identifies the LCa stages when integrated with machine learning. AUC values of 0.950 and 0.956 are obtained for discovery and validation sets, respectively, across the five‐group classification by construction a 23‐metabolite diagnostic panel. This study not only introduces a rational heterojunction design strategy for LDI‐MS use but also establishes a robust, cost‐effective analytical platform bridging nanomaterials with clinical diagnostics, thereby providing a pathway toward metabolomics‐driven precision medicine for respiratory diseases.

## Graphical abstract

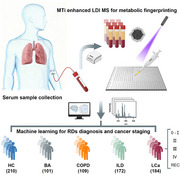

## 1 Introduction

Respiratory diseases (RDs), including bronchial asthma (BA), chronic obstructive pulmonary disease (COPD), interstitial lung disease (ILD), and lung cancer (LCa), represent growing global public health challenges [1, 2, 3, 4]. Current statistics reveal that hundreds of millions of individuals are affected by BA and COPD, more than 10 million live with ILD, and LCa is responsible for millions of deaths annually [5]. A key clinical difficulty stems from the frequent overlap of symptoms across these diseases, including wheezing, dyspnea, and coughing, often resulting in delayed diagnosis and treatment, which in turn exacerbates disease progression and worsens patient outcomes [6, 7]. Existing diagnostic approaches for RDs remain suboptimal. BA, COPD, and ILD are heterogenous respiratory disorders, whose accurate identification usually requires integrating the medical history of the patient, physical examination, pulmonary function tests, imaging, and therapeutic trials [1]. Low‐dose helical computed tomography is the primary screening method for LCa; however, its high cost and need for specialized operators limit its widespread use, and definitive diagnosis still requires invasive lung biopsy [4]. Overall, RDs diagnosis is complex and lacks a single universally accepted gold standard. Although molecular phenotyping offers a promising alternative, current methodologies lack the high throughput and cost‐effectiveness required for its application in clinical translation [8].

Accurate phenotyping and early RD diagnosis require identifying differentially expressed molecules [9, 10, 11, 12]. Although numerous candidate biomarkers have been proposed for COPD, ILD, BA, and LCa, high disease heterogeneity, with diverse etiologies and clinical trajectories, has impeded their application into routine clinical use, with only a limited number of biomarkers being successfully adopted to date [13]. Metabolomics provides direct pathological states readouts, as metabolites dynamically mirror the underlying physiological and disease processes [14, 15, 16, 17, 18]. Constructing metabolic fingerprints or feature panels helps minimize data redundancy and improve diagnostic specificity [19, 20, 21, 22, 23, 24, 25, 26], making this approach particularly suitable for distinguishing RDs. Biofluid‐based metabolomics is especially attractive owing to its noninvasive nature; however, conventional chromatography‐mass spectrometry (MS) methods are typically slow and operationally complex [27, 28, 29]. Consequently, a clear demand exists for advanced metabolic profiling tools that simultaneously exhibit high‐throughput capability, high sensitivity, and clinical scalability.

Laser desorption/ionization mass spectrometry (LDI‐MS) is an effective technique that enables high‐throughput analysis with minimal sample consumption and straightforward preparation protocols [30, 31, 32, 33, 34, 35, 36, 37, 38, 39]. The matrix, which mediates the absorption of ultraviolet laser energy, charge transfer to analytes, and thermal desorption, is a critical determinator of LDI‐MS performance. Recent nanomaterial advances have led to the development of matrices that offer superior performance to conventional organic matrices, thereby facilitating the direct metabolic profiling of complex biological samples [40, 41, 42, 43, 44, 45, 46, 47]. Among them, TiO_2_ has been widely explored owing to its favorable properties. As an *n*‐type semiconductor, TiO_2_ is endowed with a bandgap that is aligned with the energies of UV lasers commonly used in LDI‐MS, thereby enabling the efficient generation of photoinduced electron–hole pairs for analyte ionization [48, 49, 50]. Moreover, TiO_2_ is cost‐effective and chemically stable, which allows its use in practical applications. However, TiO_2_ faces critical limitations, including rapid photoinduced charge carriers recombination, which compromises ionization efficiency [51], while its weak photothermal effect hampers analyte desorption [52]. Furthermore, TiO_2_ exhibits limited tolerance to the high salt and protein content typically associated with biofluids, which restricts its use in large‐scale clinical screening scenarios [53]. Heterojunction construction has been proposed as an effective strategy for overcoming these shortcomings. MOFs, which are known for their tunable syntheses and structural versatility, are ideal templates for engineering metal‐oxide heterostructures [54, 55, 56]. MOF‐guided structural engineering affords porous, large‐surface‐area architectures with rough surfaces that enhance laser absorption and introduce a size‐exclusion effect that improves compatibility with complex biofluids [57, 58]. In addition, the built‐in electric field at the heterojunction interface suppresses charge recombination, thereby promoting metabolite ionization [59]. Despite these advantages, MOF‐derived metal oxide/TiO_2_ nanocomposites have not been thoroughly explored for LDI‐MS use, and their potential for RD metabolic profiling remains largely untapped.

**SCHEME 1:**
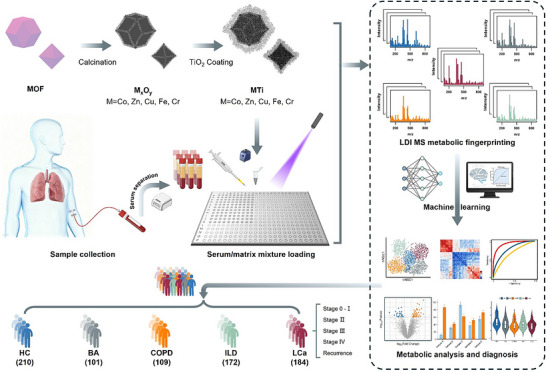
Schematic illustration of the integrated workflow for MOF‐derived heterojunction preparation, serum metabolic fingerprinting, and machine learning‐based diagnosis of respiratory diseases.

Herein, we introduce a high‐performance biofluid metabolic analysis platform based on a MOF‐derived metal oxide/TiO_2_ heterojunction (referred to as “MTi”) matrix (Scheme 1). A series of candidates, including ZnTi, FeTi, CuTi, and CrTi, was evaluated, and Co_3_O_4_/TiO_2_ (CoTi) was identified as the optimal matrix. Compared to pure TiO_2_ or Co_3_O_4_, CoTi was found to enhance metabolite signal intensities by nearly an order of magnitude. Moreover, we conducted CoTi‐assisted LDI‐MS to acquire serum‐metabolic profiles from 776 subjects, including healthy controls (HCs) and patients with BA, COPD, ILD, and LCa at different stages. Integrating machine learning into this approach led to the accurate discrimination of RDs and LCa stages. A 23‐metabolite diagnostic panel was constructed that yielded an AUC of 0.950 for the discovery set and 0.956 for the validation set. This study not only introduces a MOF‐derived heterojunction design that surmounts the inherent limitations of TiO_2_‐based matrices but also establishes a robust, high‐throughput, cost‐effective LDI‐MS platform for RD phenotyping. Our work paves the way for the use of nanomatrix‐enhanced LDI‐MS in clinical precision‐medicine applications, thereby bridging the gap between materials science and RD diagnostics.

## 2 Results and Discussion

### 2.1 Material Preparation and Characterization

**FIGURE 1:**
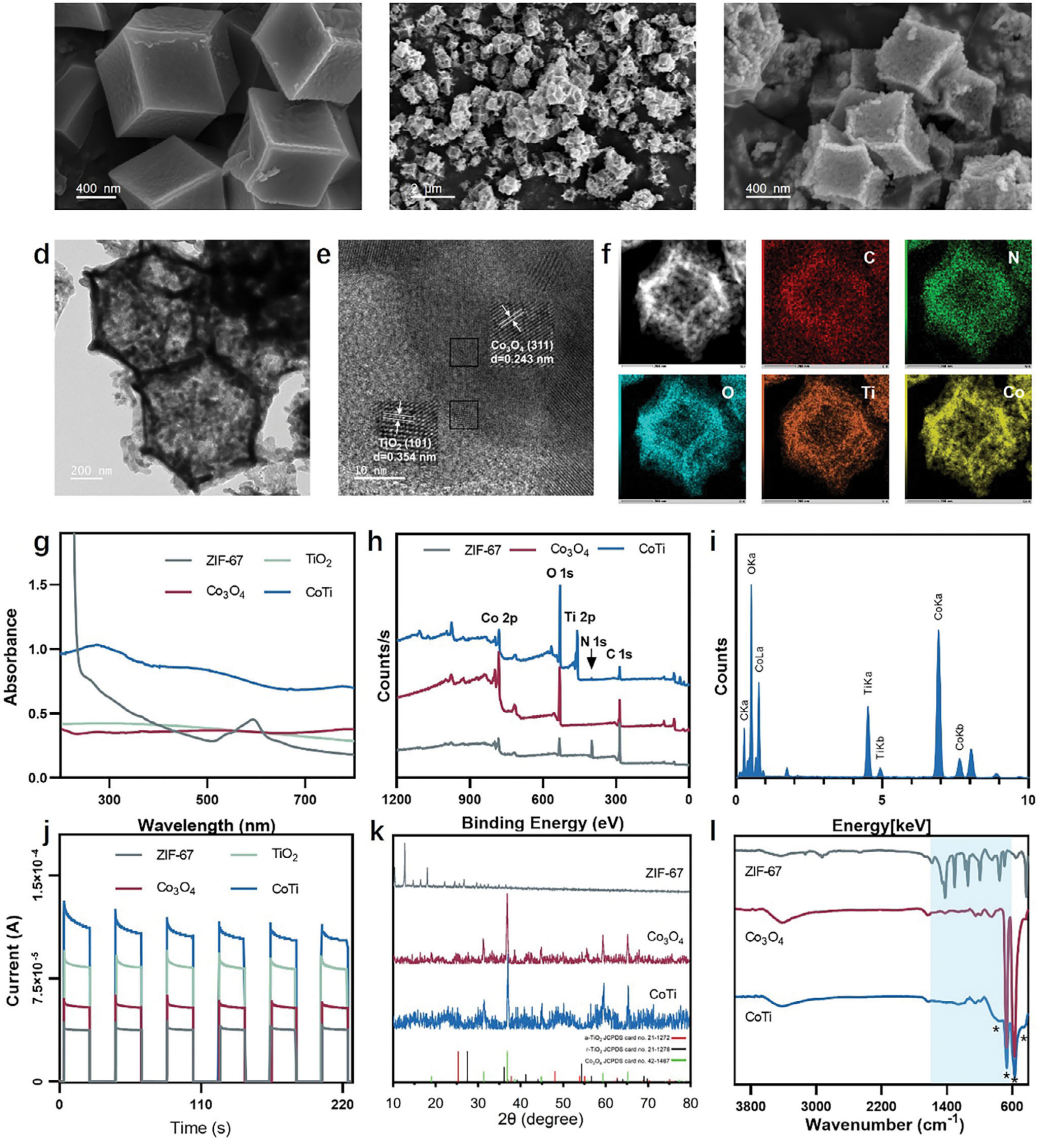
Material characterization of ZIF‐67, Co_3_O_4_, and Co_3_O_4_/TiO_2_ (CoTi). SEM images of (a) ZIF‐67, (b) Co_3_O_4_, and (c) CoTi. (d) TEM images of CoTi. (e) High‐resolution TEM image of CoTi, with lattice fringes corresponding to the (311) plane of Co_3_O_4_ and the (101) plane of anatase TiO_2_. (f) EDS elemental mapping of CoTi showing the uniform distribution of C (red), N (green), O (blue), Ti (orange), and Co (yellow). (g) UV–vis absorption spectra of ZIF‐67, TiO_2_, Co_3_O_4_, and CoTi. (h) XPS spectra of ZIF‐67, Co_3_O_4_, and CoTi. (i) EDS spectrum of CoTi. (j) Transient photocurrent response analyses of ZIF‐67, TiO_2_, Co_3_O_4_, and CoTi. (k) XRD patterns of ZIF‐67, Co_3_O_4_, and CoTi. (l) FT‐IR spectra of ZIF‐67, Co_3_O_4_, and CoTi.

The morphological and structural properties of the prepared CoTi heterostructure and other MTi composites (ZnTi, FeTi, CrTi, and CuTi) were first characterized using electron microscopy and x‐ray diffraction (XRD). As shown in Figure 1k, the XRD pattern of CoTi exhibits distinct diffraction peaks indexed to Co_3_O_4_ (JCPDS card no. 42–1467), confirming its successful derivation from ZIF‐67 following calcination. Peaks corresponding to anatase TiO_2_ (JCPDS card no. 21–1272) are also present, albeit with relatively low intensities, which can be attributed to the low content and potentially poor crystallinity of TiO_2_ within the hollow Co_3_O_4_/TiO_2_ composite. Similarly, the XRD patterns of ZnTi, FeTi, CrTi, and CuTi (Figure S2) confirm the formation of ZnO (JCPDS no. 89‐0074), Fe_2_O_3_ (JCPDS no. 89–0597), Cr_2_O_3_ (JCPDS no. 38–1479), and CuO (JCPDS no. 89–1268), respectively. The variations in TiO_2_ peak intensity and crystallinity among the different MTi materials are closely tied to their specific synthesis parameters and final compositions. Scanning electron microscopy (SEM) images reveal that the precursor ZIF‐67 particles (Figure 1a) possess well‐defined cubic morphology, indicative of high crystallinity. Following calcination and TiO_2_ coating, the resulting CoTi composites (Figure 1c) maintain the original rhombic dodecahedral framework but display roughened, partially collapsed concave surfaces with sharp corners and curled edges. This morphological evolution is attributed to the thermal decomposition of ZIF‐67 into Co_3_O_4_, which partially preserves the parent architecture, followed by the deposition of TiO_2_ without complete structural disruption. In contrast, SEM analyses of ZnTi, FeTi, CrTi, and CuTi revealed varying degrees of structural degradation in their respective MOF precursors (Figure S3), suggesting that the synthesis conditions induced more significant framework alterations. Transmission electron microscopy (TEM) further elucidates the hollow architecture of the Co_3_O_4_ skeleton and the conformal outer TiO_2_ coating layer (Figure 1d). High‐resolution TEM (HRTEM, Figure 1e) shows clear lattice fringes with spacings of 0.243 and 0.354 nm, corresponding to the (311) plane of Co_3_O_4_ and the (101) plane of anatase TiO_2_, respectively. These align well with the XRD peaks at 36.9° and 25.3°. Critically, at the interface, the adjacent (311) plane of Co_3_O_4_ and the (101) plane of TiO_2_ form a heterojunction, which is anticipated to facilitate charge transfer—a highly desirable property for an LDI‐MS matrix. Energy‐dispersive x‐ray spectroscopy (EDS) elemental mapping (Figure 1f,i) confirms the homogeneous distribution of C, N, O, Ti, and Co throughout the CoTi structure, providing conclusive evidence for the coexistence of Co_3_O_4_ and anatase TiO_2_.

The UV–vis absorption profiles of the five MTi composites are critical, given the use of a 355 nm laser in LDI‐MS. CoTi demonstrated the strongest absorption at approximately 355 nm (Figure S1), which favors efficient laser energy capture. This efficient photon absorption is essential for driving the desorption and ionization of small metabolite molecules in serum. In comparison, ZnTi showed moderate absorption, FeTi and CuTi exhibited lower intensities, and CrTi displayed a pronounced decrease in absorption near this key wavelength. The enhanced absorption of CoTi relative to its precursor (Figure 1g) underscores its superior capability to capture and transfer laser energy to analytes, thereby promoting their ionization. The strong absorption at 355 nm leads to a shallower optical penetration depth, which confines the laser energy to a localized surface volume, promoting a rapid thermal confinement effect that is highly beneficial for explosive desorption.

X‐ray photoelectron spectroscopy (XPS) was employed to investigate the chemical states and interfacial interactions in ZIF‐67, Co_3_O_4_, and CoTi (Figure 1h). In the Co 2p spectrum of ZIF‐67, the binding energies at 781.2 eV (Co 2p_3_/_2_) and 796.5 eV (Co 2p_1_/_2_) are characteristic of Co^2^^+^ within the MOF framework (Figure S4). Following calcination to form Co_3_O_4_, the Co 2p_3_/_2_ peak shifts to 780.3 eV, indicative of the mixed Co^2^^+^/Co^3^^+^ oxidation states in the spinel oxide (Figure S5). Notably, in the CoTi heterostructure, the Co 2p peaks undergo a further negative shift to 779.5 and 794.8 eV (Figure S6), signaling an increased electron density around the Co atoms—a direct consequence of the p‐n heterojunction formation between p‐type Co_3_O_4_ and n‐type TiO_2_ [60]. In the O 1s spectrum, the peak at 529.7 eV is assigned to lattice oxygen (O^2^^−^) in metal‐oxygen bonds (Co─O, Ti─O), while the peaks at 530.5 and 531.7 eV correspond to surface‐adsorbed oxygen species [61]. The Ti 2p spectrum shows binding energies at 458.6 eV (Ti 2p_3_/_2_) and 464.4 eV (Ti 2p_1_/_2_), confirming the presence of Ti^4^^+^ in anatase TiO_2_, which is consistent with the XRD results.

Fourier‐transform infrared (FT‐IR) spectroscopy provides additional evidence for the material transformations (Figure 1l). The spectrum of ZIF‐67 shows prominent bands in the 600–1500 cm^−^^1^ region, characteristic of the 2‐methylimidazole linker. These bands vanish completely after calcination, confirming the thorough decomposition of the organic framework. The resulting Co_3_O_4_ spectrum displays new absorption peaks at 665 and 567 cm^−^^1^, assigned to the stretching vibrations of Co^2^^+^─O and Co^3^^+^─O bonds in the spinel structure, respectively [62]. In the CoTi spectrum, two additional peaks emerge at 457 and 750 cm^−^^1^, which are attributed to Ti–OH stretching vibrations, confirming the successful coating of TiO_2_ onto the Co_3_O_4_ surface [63]. The persistence of the Co–O vibration bands, albeit with attenuated intensities due to the TiO_2_ overlay, verifies that the core Co_3_O_4_ structure remains intact throughout the modification process. The N_2_ adsorption–desorption isotherms of the CoTi nanomatrix confirm the presence of a hierarchical mesoporous structure formed by the aggregation of nanocrystals, a structural characteristic that facilitates the rapid diffusion and enrichment of small‐molecule metabolites.

### 2.2 Evaluating the MTi Nanomatrix for Metabolites Analysis by LDI‐MS

**FIGURE 2:**
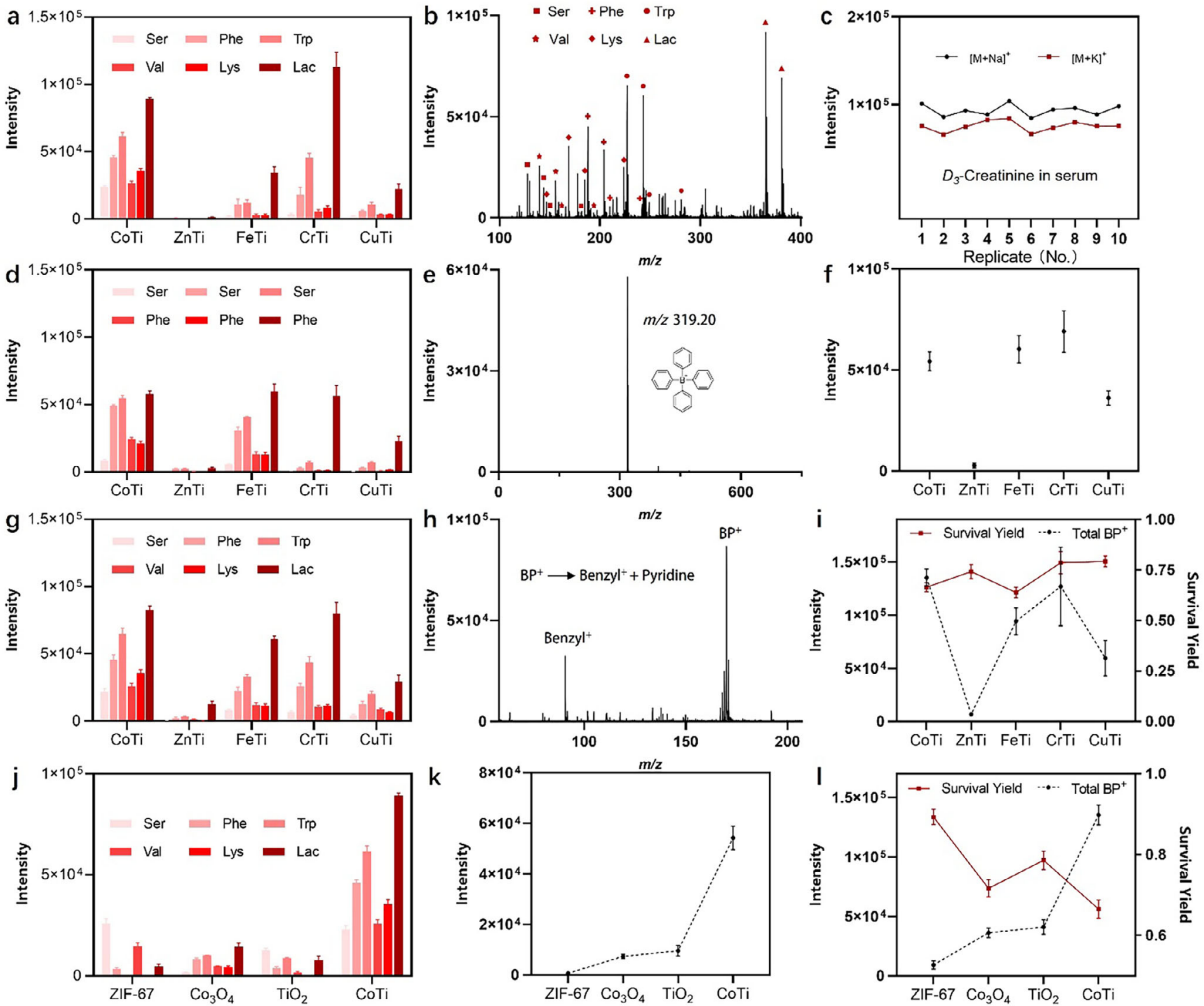
Evaluation of LDI‐MS performance using MTi nanomatrix. (a) Signal intensities of a standard metabolite mixture (Ser, Phe, Trp, Val, Lys, Lac) detected by different MTi matrices, including CoTi, ZnTi, FeTi, CrTi, and CuTi. (b) Representative mass spectrum of the metabolite mixture obtained with the CoTi matrix. (c) Spot‐to‐spot reproducibility assessed using D_3_‐creatinine spiked in serum. (d) Metabolic signals detected in 1 m NaCl solution. (e) Representative mass spectrum of the thermometric molecule TPB^−^ acquired with CoTi in negative ion mode. (f) Comparison of TPB^−^ signal intensities for different matrices. (g) Metabolic signals detected in 5 mg mL^−^^1^ BSA solution. (h) Representative mass spectrum of the thermometric molecule BP^+^ and its fragment acquired with CoTi in positive ion mode. (i) BP^+^ survival yield (SY) for different matrices. (j) Comparison of metabolic signal intensities between CoTi and its precursors. (k) TPB^−^ signal intensities of CoTi and its precursors. (l) BP^+^ survival yield of CoTi and its precursors. All quantitative data are presented as the mean ± standard deviation (SD) from n = 3–5 independent technical replicates.

The performance of the MTi nanomatrix in LDI‐MS for small‐molecule metabolite analysis was systematically evaluated, and the underlying desorption‐ionization mechanisms were probed through UV–vis spectroscopy, photocurrent response, and photothermal conversion measurements. First, the intrinsic background signals of the MTi matrices were assessed. All five MTi composites exhibited negligible background interference in the critical low‐mass range (< 500 Da), with only minor signals attributable to the target plate itself being observed (Figure S7). A standard metabolite mixture comprising serine (Ser), phenylalanine (Phe), tryptophan (Trp), valine (Val), lysine (Lys), and lactose (Lac) was then used to comparatively evaluate the detection capabilities of CoTi, ZnTi, FeTi, CrTi, and CuTi. As summarized in Figure 2a, CoTi and CrTi generated the highest overall signal intensities for the small molecules. Notably, CrTi showed an exceptionally strong response for Lac but comparatively weaker signals for amino acids such as Ser, Val, and Lys. In contrast, CoTi demonstrated consistently high signal intensities across all six diverse analytes, indicating its broader applicability compared to other MTi composites. A representative mass spectrum acquired using CoTi (Figure 2b) revealed that metabolites primarily formed [M+Na]^+^ and [M+K]^+^ adducts, alongside other ions such as [M+H]^+^, [M+2Na‐H]^+^, and [M+2K‐H]^+^. This diversity of ion adducts enhances the detection probability and confidence in metabolite identification.

Salt and protein tolerance are critical parameters governing the practical utility of a matrix in complex biofluid analysis [64, 65]. To evaluate this, the metabolite mixture was prepared in 1 m NaCl (simulating high‐salt conditions) and 5 mg/mL BSA in PBS (simulating protein‐rich environments). Under high‐salt conditions (Figure 2d), CoTi demonstrated remarkable signal stability, whereas FeTi, despite showing significant signal enhancement, still yielded lower absolute intensities than CoTi. CrTi exhibited severe signal suppression, revealing its poor salt tolerance and limited suitability for practical bioanalysis. ZnTi showed negligible signal enhancement, demonstrating inadequate performance for biofluid analysis. In the presence of high protein concentrations (Figure 2g), all MTi matrices performed better than in high‐salt environments. CoTi, FeTi, and CrTi produced intense and comparable signals for all metabolites, while ZnTi and CuTi also showed measurable signal enhancement. This performance is attributed to the porous nanostructure of the MTi matrices, which can selectively entrap small metabolite molecules while excluding protein macromolecules, thus mitigating suppression effects. Furthermore, the low‐concentration salt ions present in the PBS buffer (0.05 m) facilitated the formation of sodium/potassium adducts, thereby enhancing metabolite ionization.

Thermo‐desorption efficiency is a key parameter for LDI‐MS nanomatrix [66, 67, 68]. The negatively charged tetraphenylborate (TPB^−^, *m/z* 319.2) and the positively charged benzylpyridinium (BP^+^, *m/z* 170.1) were employed as thermometer molecules in negative and positive ion modes, respectively. In negative mode (Figure 2e,f), CoTi, FeTi, CrTi, and CuTi generated intense TPB^−^ signals, indicative of effective thermal desorption, whereas ZnTi showed a markedly lower response. CoTi, in particular, produced a stable TPB^−^ signal with minimal background (Figure 2e). In positive ion mode, BP^+^ undergoes a C─N bond cleavage when the energy transferred from the matrix exceeds its critical dissociation energy, yielding a benzyl^+^ fragment (*m/z* 91.1) and neutral pyridine (Figure 2h). The survival yield (SY), defined as the ratio of the parent BP^+^ intensity to the total ion signal (parent + fragment), is inversely related to the transferred thermal energy. As shown in Figure 2i, CoTi (66.5%) and FeTi (63.8%) exhibited the lowest SY values, significantly lower than those of ZnTi (74.2%), CrTi (78.6%), and CuTi (79.3%). Although the SY values of thermometer ions only provide a comparative evaluation rather than absolute physical plume temperatures, they serve as robust and widely accepted metrics to confirm the enhanced photothermal conversion and energy transfer efficiencies of the LDI matrix [67, 68]. These results demonstrate that CoTi and FeTi possess the highest thermal desorption efficiency among the series, a critical factor for sensitive LDI‐MS analysis.

Charge separation efficiency, governed by the heterojunction interface, is another pivotal factor determining ionization performance [69, 70]. Transient photocurrent responses were measured to evaluate this property. As depicted in Figure S1b, among all MTi composites, CoTi demonstrated the highest photocurrent density, underscoring its superior charge separation capability. While FeTi also exhibited considerable thermal desorption performance, its relatively lower charge separation efficiency limits its overall LDI‐MS performance. Moreover, CoTi exhibited a photocurrent density nearly double that of pristine Co_3_O_4_ (Figure 1j), while ZIF‐67 showed a minimal response. Mott–Schottky (M–S) electrochemical measurements were performed to verify the semiconductor types. As shown in Figure S20, the positive slope of the TiO_2_ plot and the negative slope of the Co_3_O_4_ plot explicitly confirm their *n*‐type and *p*‐type semiconducting characteristics, respectively. This empirically validates the successful construction of the *p–n* heterojunction. This dramatic enhancement unambiguously confirms that the formation of the *p–n* heterojunction between Co_3_O_4_ and TiO_2_ effectively promotes the separation of photogenerated electron‐hole pairs and suppresses their recombination. This result is directly consistent with the superior LDI‐MS signal enhancement observed for CoTi, establishing a direct correlation between improved charge separation and enhanced analytical performance in LDI‐MS.

Matrix spot homogeneity is crucial for ensuring signal reproducibility. Images of dried matrix‐analyte droplets (Figure S8) revealed that ZnTi, CrTi, and CuTi spots suffered from inhomogeneous distribution or coffee‐ring effects. In contrast, CoTi and FeTi formed uniform, homogenous spots, which directly contributed to their lower signal relative standard deviations (RSDs), confirming excellent spot‐to‐spot reproducibility. Profiling of human serum (Figure S11) revealed that ZnTi and CuTi were largely ineffective, whereas CoTi generated rich metabolic fingerprints with low background, with CoTi yielding the most comprehensive profiles. Based on this evaluation, CoTi was identified as the optimal nanomatrix and selected for subsequent biofluid analysis. The universality of CoTi was further validated by its successful application to other biofluids, including sweat, urine, saliva, and tears, confirming its broad applicability (Figure S12).

The performance of the CoTi heterostructure was then compared with its precursors (Co_3_O_4_, TiO_2_, and ZIF‐67). As shown in Figure 2j, CoTi generated intense signals for all metabolites, with sodium adduct signals stronger than potassium adducts (Figure S9c). In contrast, Co_3_O_4_ and TiO_2_ produced only weak signals, and ZIF‐67 failed to detect Trp and Lys, underscoring its insufficient sensitivity. In salt and protein tolerance tests (Figure S9a, b), CoTi maintained stable, high‐intensity signals, outperforming its precursors. In thermal desorption tests (Figure 2k,l), the performance of CoTi was markedly superior, with its desorption efficiency in negative ion mode approximately 6 and 7 times greater than that of TiO_2_ and Co_3_O_4_, respectively, far exceeding the sum of their individual contributions. Similarly, in positive ion mode, its signal intensity was about 4‐fold higher than that of either precursor. The SY of CoTi (66.5%) was also lower than that of TiO_2_ (78.6%), Co_3_O_4_ (71.7%), and ZIF‐67 (89.3%). The robust survival yield evaluation, alongside the photocurrent responses, highlights the synergistic nature of photothermal desorption and photochemical ionization in driving the superior LDI‐MS performance of the CoTi heterojunction. Optimization of the matrix concentration revealed that signal intensity increased with CoTi concentration from 0.5 to 5 mg/mL but decreased at 10 mg/mL (Figure S9d), identifying 5 mg/mL as the optimal balance for efficient LDI, thus establishing it as the standard concentration for subsequent experiments. The reproducibility and quantitative capability of CoTi were further validated. Linear correlations (R^2^ ≈ 0.99) were observed between the signal intensities and concentrations for all six standard metabolites (Figure S10). For a practical reproducibility assessment, *D_3_*‐creatinine (5 mM) was spiked into serum, and measurements across over ten independent spots showed consistent signals with RSDs of 6.75% and 6.89% for [M+Na]^+^ and [M+K]^+^, respectively (Figure 2c). These results collectively confirm the outstanding potential of CoTi as a robust and high‐performance nanomatrix for the LDI‐MS of biofluid analysis. From a translational perspective, the CoTi‐LDI‐MS platform offers practical advantages over traditional LC‐MS clinical methods. By eliminating the need for time‐consuming chromatographic separation, the data acquisition time is reduced from 15–30 min per sample in LC‐MS to mere seconds per spot in LDI‐MS. Furthermore, consumable costs are drastically minimized, as the method operates without expensive solvents or costly columns. The synthesized CoTi matrix is highly cost‐effective and is consumed only in microgram quantities per assay. These time and cost efficiencies make this platform highly viable for routine, high‐throughput clinical screening.

### 2.3 Metabolic Profiling RDs from HC

**FIGURE 3:**
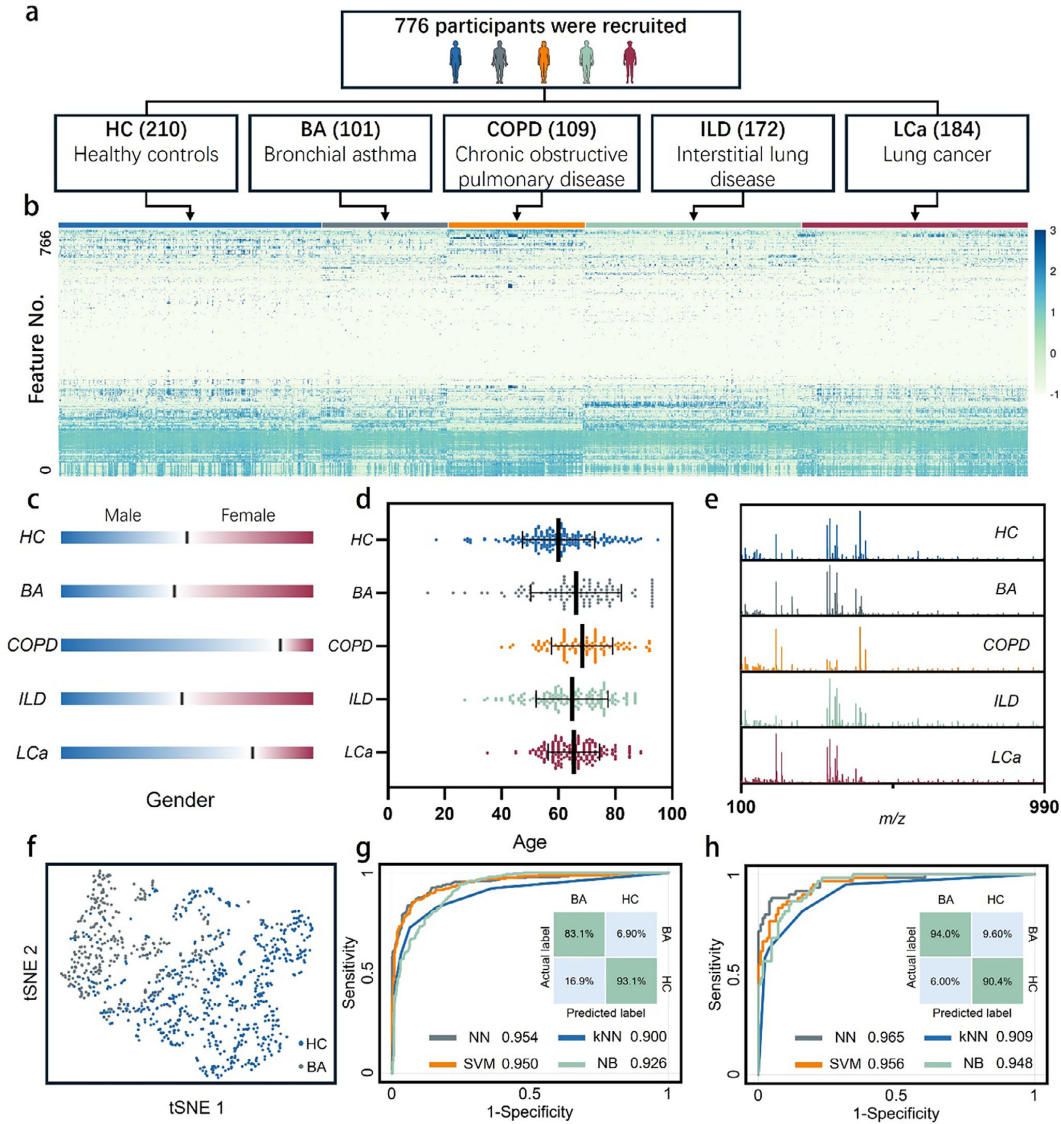
(a) Cohort distribution of 776 participants., including healthy control (HC, n = 210), bronchial asthma (BA, n = 101), chronic obstructive pulmonary disease (COPD, n = 109), Interstitial lung disease (ILD, n = 172), and lung cancer (LCa, n = 184). (b) The heatmap of the extracted serum metabolic fingerprints from 776 individuals. (c) Gender and (d) Age distribution of the recruited cohort. (e) Representative mass spectra from each group. (f) t‐SNE plot of HC vs. BA based on the top 10 m/z feature panel. ROC curves for discriminating BA from HC in the (g) discovery (n = 168 for HC; n = 81 for BA) and (h) validation sets (n = 42 for HC; n = 20 for BA) using the top 10 *m/z* feature panel.

Respiratory diseases such as BA, COPD, and ILD are heterogeneous conditions involving varying degrees of inflammation and airway damage [71]. They commonly present with overlapping clinical symptoms such as wheezing, dyspnea, and coughing, which complicate differential diagnoses in the absence of a universally accepted gold standard. Meanwhile, LCa often remains asymptomatic in its early stages, leading to delayed treatment and underscoring the critical need for early screening. The CoTi‐assisted LDI‐MS platform offers a promising solution due to its high throughput, low cost, and noninvasive nature, making it well‐suited for rapidly extracting metabolic phenotypes and enabling accurate disease discrimination. We collected 776 serum samples from healthy individuals (control samples) and patients diagnosed with BA, COPD, ILD, or different stages of LCa (Figure 3a). Participant demographics, including age and gender distribution, are summarized in Figure 3c,d, and Table S1. Consistent with epidemiological trends, the incidences of COPD and LCa were found to be significantly higher in men than in women. Representative mass spectra from each group revealed visible spectral differences, with most intense signals observed below *m/z* 500. Serum metabolic fingerprints were acquired for all samples, which led to the generation of a data matrix comprising 766 *m/z* features and 2298 spectra (Figure 3b). The data matrix was clustered according to the peak extraction, alignment, normalization, and standardization, which revealed clear global differences between groups. This separation underscores both the analytical performance of the CoTi matrix and the underlying metabolic distinctions among the five cohorts.

**FIGURE 4:**
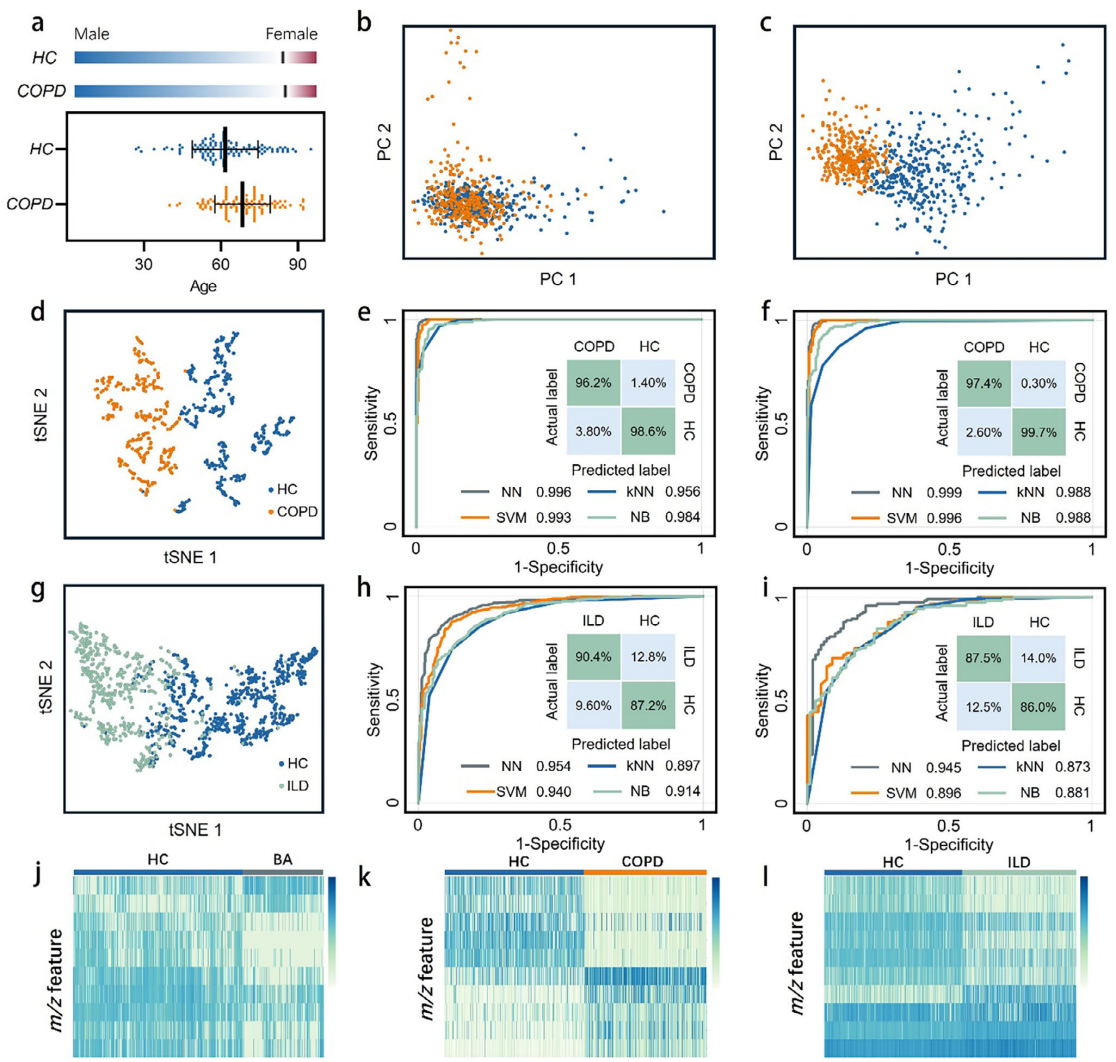
(a) Gender and age distribution of matched HC (n = 122) and COPD (n = 109) groups. (b) PCA plot of HC vs. COPD using the full metabolic spectrum. (c) PCA and (d) t‐SNE plots of HC vs. COPD using the top 10 m/z feature panel. ROC curves for discriminating COPD from HC in the (e) discovery set (n = 98 for HC; n = 87 for COPD) and (f) validation set (n = 24 for HC; n = 22 for COPD) using the feature panel. (g) t‐SNE plot of HC vs. ILD using the top 10 m/z feature panel. ROC curves for discriminating ILD from HC in the (h) discovery set (n = 168 for HC; n = 138 for ILD) and (i) validation set (n = 42 for HC; n = 34 for ILD) using the feature panel. Heatmaps of the top 10 key m/z features for (j) BA, (k) COPD, and (l) ILD classification.

We first aimed to discriminate BA patients from HCs using serum metabolic fingerprints, which involved analyzing 311 samples comprising 210 HC and 101 BA samples. PCA revealed a substantial overlap between the two groups, although the observed data‐point clustering is consistent with high analytical reproducibility (Figure S13a). t‐SNE also failed to provide clear separation (Figure S13e), confirming that conventional dimensionality‐reduction methods insufficiently distinguish BA from HC. Therefore, we employed multiple machine learning classifiers—Neural Networks (NN), Support Vector Machines (SVM), K‐Nearest Neighbors (KNN), and Naïve Bayes (NB)—using an 80/20 split for the discovery and validation sets. All four classifiers performed consistently well on the discovery set (Figure S13b; Table S2), with NN, SVM, KNN, and NB delivering AUC values of 0.973, 0.983, 0.980, and 0.923, respectively. The corresponding confusion matrix revealed high classification accuracies, with values of 95.9% and 96.7% recorded for BA and HC, respectively. These results were robustly validated using the independent validation set (Figure S13c; Table S3), where the various models maintained high AUCs (NN: 0.989; KNN: 0.969; SVM: 0.996; NB: 0.917) and superior accuracies (BA: 100%; HC: 97.7%). A volcano plot (Figure S13f) further substantiated these findings, identifying 123 downregulated and 72 upregulated *m/z* features with significant differences, thereby providing a molecular basis for the observed metabolic divergence. We constructed a condensed diagnostic panel to improve model efficiency and clinical translatability by selecting the top 10 discriminative *m/z* features according to the classifier ranking score (Figure 4j; Table S4). Both PCA (Figure S13d) and t‐SNE (Figure 3f) showed markedly improved separation between BA and HC groups using this refined panel. All models continued to perform strongly in the discovery set when evaluated using the top‐10 feature set (Figure 3g; Table S5), with AUC of 0.954 (NN), 0.900 (KNN), 0.950 (SVM), and 0.926 (NB), and accuracies of 83.1% for BA and 93.1% for HC. The validation set (Figure 3h; Table S6) confirmed these results, with AUC values of 0.965 (NN), 0.909 (KNN), 0.956 (SVM), and 0.948 (NB), and classification accuracy of 94.0% (BA) and 90.4% (HC). These outcomes underscore the effectiveness of the selected metabolic panel for accurately distinguishing patients with BA from healthy individuals.

COPD is increasingly regarded as a complex syndrome rather than a single disease entity, given its heterogeneous clinical presentation and multifactorial pathophysiology [72]; consequently, elucidating its metabolic phenotype can significantly aid in its clinical identification [8]. To ensure valid group comparisons, we selected a gender‐ and age‐matched subset from the HC group, which resulted in 122 individuals (107 male and 15 female) for comparison with COPD patient cohort (Figure 4a; Table S7). Although PCA showed limited separation (Figure 4b), t‐SNE analysis revealed a clearer trend toward group discrimination (Figure S14a). A volcano plot revealed numerous differentially expressed metabolites (Figure S14d), showing that the metabolic‐fingerprint‐based diagnosis is feasible. Machine learning models trained on the full metabolic data exhibited excellent performance for the discovery set (Figure S14b; Table S8), with near‐perfect AUC values determined for NN (0.986), KNN (0.999), SVM (0.990), and NB (0.994). The confusion matrix showed high classification accuracies (COPD: 96.2%; HC: 97.9% for actual labels vs predicted labels). These results were strongly replicated for the validation set (Figure S14c; Table S9), in which all models delivered near‐perfect discrimination. We subsequently constructed a 10‐*m/z* features panel (Figure 4k; Table S10) to enhance diagnostic efficiency, which led to both PCA and t‐SNE exhibiting clear group separation (Figure 4c, d). Machine learning models maintained high performance for the discovery set (Figure 4e; Table S11), with AUC values above 0.95 for all classifiers, while the confusion matrix showed high accuracies (COPD: 96.2%, HC: 98.6%). The validation set (Figure 4f; Table S12) confirmed these results: AUC values exceeded 0.988, and the confusion matrix also revealed high classification accuracies (COPD: 97.4%; HC: 99.7%), confirming the clinical potential of this metabolic panel for use in COPD‐screening applications.

Interstitial lung disease is a common respiratory disorder characterized by impaired gas exchange, leading to dyspnea, cough, and diminished quality of life. Idiopathic pulmonary fibrosis, its most common form, carries a poor prognosis, with a median survival of 3–5 years post‐diagnosis. Accurate diagnosis is therefore critical but remains challenging due to non‐specific clinical presentations, often necessitating a multidimensional diagnostic approach [73]. A volcano plot revealed numerous metabolites that are differentially expressed between ILD and HC (Figure S15f); while PCA showed limited separation (Figure S15a), t‐SNE revealed a discernible clustering trend (Figure S15e). Machine learning models trained on the full metabolic data performed well on the discovery set (Figure S15b; Table S13), with AUC values of 0.983 (NN), 0.959 (KNN), 0.980 (SVM), and 0.907 (NB). Performance remained strong across the validation set (Figure S15c; Table S14), particularly for NN (AUC = 0.998). A focused 10 *m/z* feature panel was then constructed (Table S15), which improved group separation for both PCA (Figure S14d) and t‐SNE (Figure 4g). The models continued to perform well for the discovery set using this panel (Figure 4h; Table S16), which showed robust validation performance (Figure 4i; Table S17), with AUC values of 0.945 (NN), 0.873 (KNN), 0.896 (SVM), and 0.881 (NB). The slight decline in performance relative to that of the full dataset suggests that while feature selection improves interpretability, some informative signals may be excluded, suggestive of a trade‐off between simplicity and predictive power.

**FIGURE 5:**
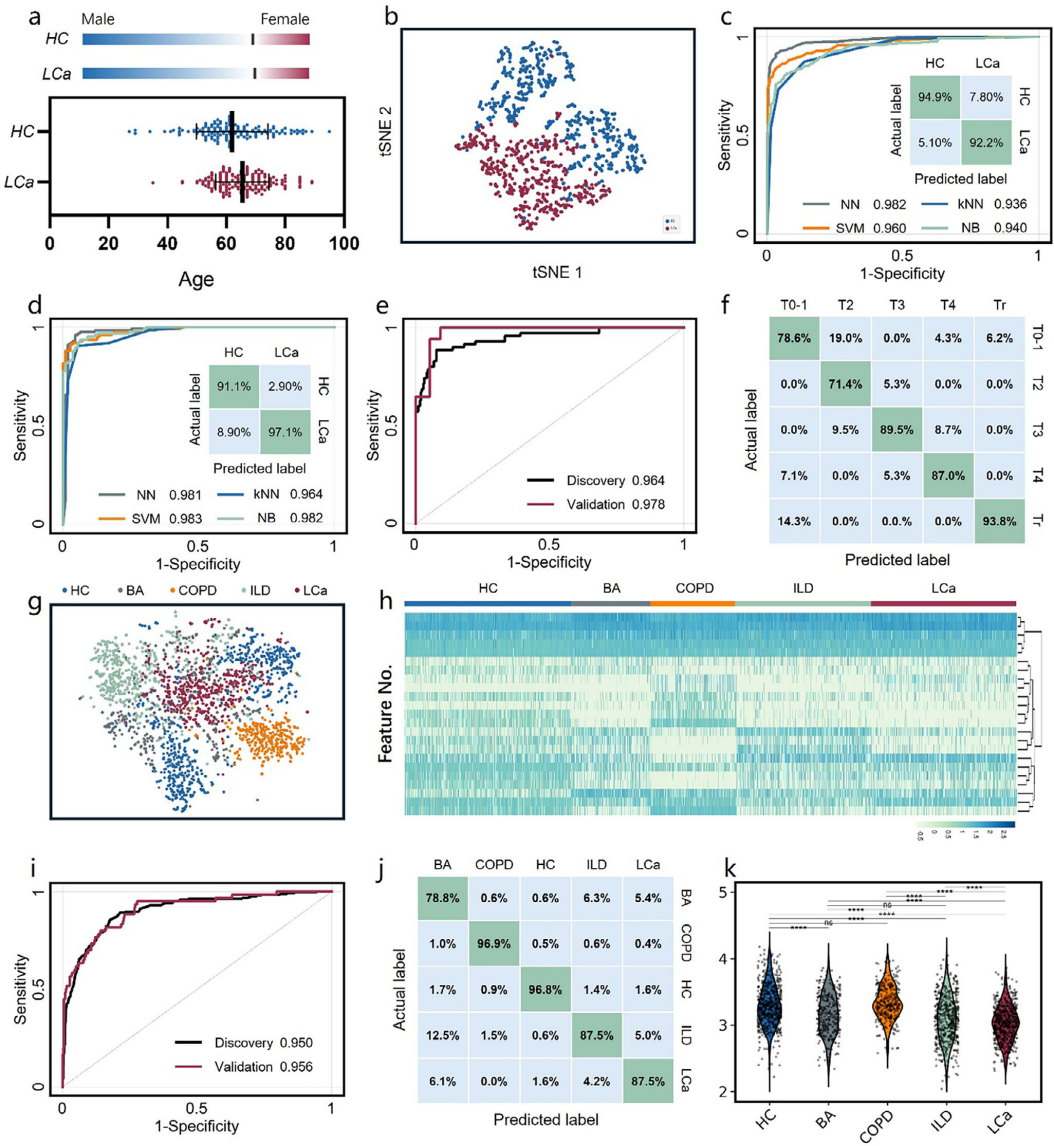
(a) Gender and age distribution of the matched HC (n = 143) and LCa (n = 184) groups. (b) t‐SNE plot and (c, d) ROC curves for discriminating LCa from HC using the top 10 m/z feature panel in the discovery (n = 114 for HC; n = 147 for LCa) and validation set (n = 29 for HC; n = 37 for LCa). (e) ROC curves and (f) confusion matrix for LCa staging using the Neural Network (NN) model (Sample size: n = 157; 27 cases were excluded from the staging analysis due to incomplete clinical stage records. n = 29 stage 0–I, n = 27 stage II, n = 35 stage III, n = 37 stage IV, and n = 29 recurrence cases). (g) t‐SNE plot and (h) heatmap of the 23‐metabolite panel for five‐group classification (HC, BA, COPD, ILD, LCa). (i) ROC curves and (j) confusion matrix for five‐group classification (Total sample size: n = 210 for HC; n = 101 for BA; n = 109 for COPD; n = 172 for ILD; and n = 184 for LCa) using the NN model and the 23‐metabolite panel. (k) Relative abundance of serum choline across different groups. Statistical significance was determined using a two‐tailed Student's *t*‐test was used for two‐group comparisons (*p*‐values were strictly adjusted using the FDR method). ^*^p < 0.05, ^**^ *p* < 0.01, ^***^ *p* < 0.001, ^**^ *p* < 0.0001, ns: not significant.

We analyzed serum samples from 327 volunteers, including 184 patients with LCa and 143 age‐ and gender‐matched HCs to evaluate the diagnostic potential of metabolic LCa fingerprinting (Figure 5a; Table S18). Machine learning models delivered high AUC values for the discovery set using the full metabolic data (Figure S16a; Table S19), with NN and KNN yielding values of 0.980, while SVM and NB delivered values of 0.960 and 0.916, respectively. The confusion matrix showed classification accuracies of 96.4% and 95.5% for HC and LCa, respectively, for actual labels vs. predicted labels. The validation set confirmed these results, with AUC values of up to 0.995 (NN), and the confusion matrix indicating high classification accuracies (HC: 97.8%; LCa: 97.3%) (Figure S16b; Table S20). A volcano plot revealed significant metabolic differences between LCa and HC groups (Figure S16d), which supports the rationality of the model. We then derived the 10 m/z diagnostic panel (Figure S16c; Table S21). Although the PCA on the full dataset indicated poor separation (Figure S16e), using the top 10 features improved clustering in both PCA (Figure S16g) and t‐SNE (Figure 5b). The model continued to perform strongly for the discovery set using the feature panel (Figure 5c; Table S22), with AUC values above 0.93 for all classifiers and the confusion matrix showing high accuracies (HC: 94.9%; LCa: 92.2%). These results were confirmed using the validation set (Figure 5d; Table S23), with NN, KNN, and SVM delivering AUC values above 0.96, and classification accuracies above 0.87, highlighting the robust diagnostic capability of the panel.

Early diagnosis and accurate staging are essential for effective LCa management. We further evaluated the ability of the metabolic fingerprints to discriminate between five LCa stages: 29 stage 0–I, 27 stage II, 35 stage III, 37 stage IV, 29 recurrence cases, and 27 cases lacked clear staging records We used full metabolic data to maximize model performance given the limited sample size per stage. The NN model exhibited outstanding performance, delivering an AUC value of 0.964 and an accuracy of 83.2% for the discovery set (Figure 5e; Tables S24, S25). These results were validated using an independent set, which revealed an AUC value of 0.978 and an accuracy of 83.9%. The confusion matrix (Figure 5f) provided stage‐specific insights, with classification accuracies of 78.6%, 71.4%, 89.5%, and 87.0% for T0–1, T2, T3, and T4, and 93.8% for recurrence. Although some misclassifications were observed, particularly for the early stages, the overall high accuracy observed across all stages highlights the potential of serum metabolic fingerprinting for LCa stage and recurrence monitoring. Future studies with larger cohorts are warranted to further improve this diagnostic strategy.

### 2.4 RD Metabolic Profiling and Metabolite Analysis

Building upon the results presented above, we validated the efficacy of the CoTi‐assisted LDI‐MS platform for extracting serum metabolic fingerprints capable of distinguishing BA, COPD, ILD, and LCa from HCs, as well as for differentiating LCa stages. We employed machine learning models to analyze a comprehensive metabolic dataset and precisely differentiate the five groups. The t‐SNE plot based on the full metabolic profile (Figure S17a) revealed a discernible clustering trend within each group and partial separation between groups, although complete distinction was not achieved. Among the classifiers evaluated in the discovery set (Figure S17b; Table S28), the NN model exhibited superior performance, with an AUC value of 0.978, outperforming SVM (0.973), kNN (0.950), and NB (0.924). The corresponding confusion matrix for the NN model (Figure S17c) revealed high classification accuracy and correctly identified 82.6% of the BA instances, with 95.5%, 95.1%, 89.2%, and 89.6% recorded for the COPD, HC, ILD, and LCa samples. This strong performance was also observed for the independent validation set (Figure S17d; Table S29), where the NN model delivered an AUC value of 0.990 and high confusion matrix performance (77.6% for BA, 95.5% for COPD, 97.5% for HC, 94.0% for ILD, and 91.8% for LCa; Figure S17e), confirming its ability to rapidly and precisely differentiation multi class of RDs. We constructed a condensed metabolite panel to improve diagnostic efficiency and enhance clinical practicality. We used a feature‐selection process based on the top 50 ranking scores, frequencies greater than 70%, and ANOVA p‐values below 0.05 to establish a 23 *m/z* feature panel for five‐group classification (Table S26). t‐SNE analysis using this refined panel (Figure 5g) exhibited superior group separation to that achieved for the full dataset, which indicates that the machine‐selected features are well aligned with metabolic variance. Heatmap visualization (Figure 5h) further confirmed the significant differences in the expression patterns of these 23 features across the groups. A comparative radar chart evaluation of the four machine learning models identified NN as the top performer across all classification tasks (Figure S18). The NN model trained on the 23‐feature panel delivered high AUC values of 0.950 and 0.956 for the discovery and validation sets, respectively. (Figure 5i, Tables S30,S31). The confusion matrix for the validation set (Figure 5j) demonstrated robust classification accuracies of 78.8%, 96.9%, 96.8%, 87.5%, and 87.5% for the BA, COPD, HC, ILD, and correctly classified LCa samples, respectively. Notably, diagnostic performance using the 23‐feature panel (Tables S30,S31) showed no significant degradation in relation to that observed using the full metabolic spectrum (Tables S28, 29), which indicates that this minimized feature set retains sufficient information to effectively discriminate between the five respiratory conditions.

The 23 metabolites in the diagnostic panel were putatively identified using a 15T FT‐ICR mass spectrometer by matching accurate mass measurements to the Human Metabolome Database with a 2 ppm mass tolerance. Nine metabolites were putatively identified, including choline, histidine, hydroxydodecanedioic acid, lysylglutamine, trimethylammoniobutanoic acid, dihydroxycinnamic acid glucuronide, hexadecanedioic acid, vanillylmandelic acid, and creatinine (Table S27). Choline plays a fundamental role in maintaining cell membrane integrity, facilitating methylation processes, and synthesizing neurotransmitters [74]. In cancer biology, rapid proliferation of malignant cells drives increased choline uptake through enzyme overexpression, transporter variation, and altered signaling pathways, leading to profound choline metabolism dysregulation [75, 76]. Consistent with this understanding, we observed the significant downregulation of serum choline in the LCa group compared to that in the HC, BA, COPD, and ILD groups (Figure 5k). This decrease in serum choline levels in NSCLC patients may reflect increased metabolic demand by highly proliferative tumor cells [77]. Histidine, an essential amino acid and a precursor for the biosynthesis of histamine and carnosine, showed distinct differences across the disease groups. The BA group exhibited moderately elevated histidine levels in relation to the HC group, while the COPD, ILD, and LCa groups exhibited significantly lower levels (Figure S19a). Histidine supplementation has been shown to exert anti‐inflammatory effects; hence, lower histidine concentrations may contribute to the pro‐inflammatory characteristic associated with COPD [78, 79]. Conversely, elevated histidine‐derived histamine levels are associated with impaired lung function, inflammatory responses, and bronchoconstriction in patients with asthma [80, 81]. These findings suggest that histidine–histamine metabolism is significantly disrupted across the spectrum of RDs. The remaining seven metabolites exhibited significant differences in abundance across the five groups. Pathway‐enrichment analysis performed using MetaboAnalyst 6.0 revealed several key metabolic pathways that are significantly associated with the identified metabolites (Figure S19i,j), with histidine metabolism being the most significantly enriched and considerably impacting pathway. Additional pathways, including beta‐alanine metabolism, the one carbon pool by folate, and lysine degradation, were also notably enriched. An overview of the top 25 enriched metabolite sets highlighted further pathways such as methylhistidine metabolism and phosphatidylethanolamine biosynthesis. These enriched pathways are critically involved in amino acid metabolism, phospholipid biosynthesis, and one‐carbon metabolism, processes that are essential for cellular energy production, signal transduction, and structural maintenance. The distinct enrichment patterns observed across the five groups are suggestive of disease‐specific metabolic reprogramming under the various respiratory conditions, which provides valuable insights into underlying molecular mechanisms and potential metabolic biomarkers.

## 3 Conclusion

We developed MOF‐derived CoTi as a high‐performance nanomatrix for use in LDI‐MS applications. The constructed p‐n heterojunction facilitated efficient charge separation and enhanced thermal desorption, leading to a significantly higher metabolite‐detection sensitivity. A high‐throughput and robust methodology for serum metabolic fingerprinting was established by integrating the CoTi‐LDI‐MS platform with machine learning. This approach demonstrated exceptional clinical utility for precisely differentiating patients with major RDs from HCs, while further discriminating LCa stages. The development of a compact 23‐feature diagnostic panel underscores the clinical translatability of our platform and provides a pathway toward rapid and cost‐effective screening. Despite the robust performance and the relatively large sample size used in this study, we acknowledge that the current model was developed and validated within a single clinical center. To further confirm the generalizability and clinical utility of this metabolic panel, rigorous multicenter external validations across diverse patient populations are warranted in our future work. Ultimately, this study highlights the immense potential of rational material design in advancing analytical science and underscores the value of metabolic phenotyping for analyzing complex RDs. It also bridges the critical gap between functional nanomaterials and practical clinical diagnostics, and provides new early disease detection and personalized medicine avenues.

## 4 Experimental Section

### 4.1 Chemicals and Reagents

Zinc acetate hydrate, cobalt nitrate hydrate, 1,2‐dimethylimidazole, titanium bis(ammonium lactato)dihydroxide (TiBALDH), and tetrabutyl titanate were purchased from Aladdin Biochemical Technology Co., Ltd. (Shanghai, China). Serine (Ser), Phenylalanine (Phe), Valine (Val), and Lysine (Lys) were obtained from Alfa Aesar (MA, USA). Tryptophan (Trp) was supplied by Sinopharm Chemical Regent CO., Ltd. (Shanghai, China). Lactose (Lac, 98%) was acquired from J&K Scientific Ltd. (Beijing, China). *D_3_*‐creatinine (> 98%) was sourced from Toronto Research Chemicals (Toronto, Canada). Sodium tetraphenylboron and 1‐benzylpyridinium chloride were purchased from Sigma–Aldrich (St. Louis, MO, USA). Bovine serum albumin (BSA) was purchased from Solarbio Science & Technology Co. Ltd. (Beijing, China). All chemicals were used as received without further purification. HKUST‐1 (CuBTC), MIL‐101(Fe), and MIL‐101(Cr) were obtained from Nanjing XFNANO Materials Tech Co.,Ltd. (XFNANO). Deionized (DI) water was used throughout the experiments.

### 4.2 Synthesis of ZIF‐67 and ZIF‐8

ZIF67 and ZIF‐8 were synthesized according to a previously reported method. Briefly, Zinc acetate hydrate or cobalt nitrate hydrate was separately mixed with 1,2‐dimethylimidazole in deionized water. The mixture was sonicated for 10 min and then aged at room temperature for 24 h. The resulting precipitate was collected by centrifugation, thoroughly washed with deionized water and ethanol, and finally vacuum‐dried at room temperature for 12 h.

### 4.3 Synthesis of MOF Derived Metal Oxide/Titanium Dioxide Heterostructure (MTi)

**CoTi**: ZIF‐67 (0.8 g) was calcined in a tube furnace by heating from room temperature to 450°C at a ramp rate of 2°C min^−1^ and maintained at this temperature for 2 h to obtain MOF‐derived Co_3_O_4_. Subsequently, 0.2 g of the as‐prepared Co_3_O_4_ was redispersed in 96 mL of dilute HCl solution (0.1 m) under stirring. Then, 4 mL of TiBALDH (50 wt.%) was added as the titanium precursor. The mixture was vigorously stirred at room temperature for 3 h, followed by centrifugation. The collected solid was washed with ethanol and deionized water and vacuum‐dried overnight at 60°C. Finally, the dried powder was calcined under a nitrogen atmosphere at 400°C for 5 h to yield the hollow‐structured Co_3_O_4_@TiO_2_ (CoTi).
**ZnTi and CuTi**: ZIF‐8 or HKUST‐1 (CuBTC) (0.1 g) was dispersed in 40 mL of ethanol under vigorous stirring. Then, 2 mL of Tetrabutyl titanate was added. The mixture was transferred to a Teflon‐line stainless‐steel autoclave and subjected to a hydrothermal reaction at 150°C for 16 h. After cooling to room temperature, the product was collected by centrifugation, washed with deionized water/ethanol, and vacuum dried at 60°C for 12 h. The powder was further calcined in air at 500°C for 3 h (ramp rate: 5°C min^−1^) to obtain ZnO@TiO_2_ (ZnTi) or CuO@TiO_2_ (CuTi).
**FeTi**: MIL‐101(Fe)(0.2 g) was dispersed in 96 mL HCl solution (0.1 m), followed by the immediate addition of 4 mL TiBALDH (50 wt.%). The mixture was stirred at room temperature for 3 h, then washed, dried, and calcined under nitrogen atmosphere at 500°C for 2 h (ramp rate: 5°C min^−1^) to obtain Fe_2_O_3_@TiO_2_ (FeTi).
**CrTi**: MIL‐101(Cr)(0.2 g) was first calcined in air at 500°C for 3 h (ramp rate: 5°C min^−1^) to yield Cr_2_O_3_. The resulting Cr_2_O_3_ was redispersed in 96 mL HCl (0.1 m), followed by the addition of 4 mL of TiBALDH (50 wt.%). The mixture was stirred for 3 h, centrifuged, washed, and vacuum‐dried at 60°C overnight to obtain Cr_2_O_3_@TiO_2_ (CrTi).

### 4.4 Materials Characterization

Transmission electron microscopy (TEM) images and energy‐dispersive x‐ray spectroscopy (EDS) data were acquired using a JEM F200 cold field emission transmission electron microscope (JEOL Ltd.). Scanning electron microscopy (SEM) was performed on a Sigma 560 hot field emission scanning electron microscope (Carl Zeiss Microscopy). UV–vis spectra were recorded on a Lambda 1050+ spectrophotometer (PerkinElmer). X‐ray photoelectron spectroscopy (XPS) was conducted on an ESCALAB 250XI system (Thermo Fisher Scientific); all the binding energies in the XPS spectra were calibrated using the adventitious C 1s peak as a charge reference. X‐ray diffraction (XRD) patterns were obtained using a D8 ADVANCE diffractometer (Bruker Corporation). Fourier‐transform infrared (FTIR) spectroscopy was performed on a Nicole iS spectrometer (Thermo Fisher Scientific). Photocurrent measurements (*i–t* curves) and Mott–Schottky (M–S) measurements were carried out on a CHI760E electrochemical workstation (Shanghai Chenhua Instruments Ltd.). N_2_ adsorption–desorption isotherms (BET measurement) were obtained at 77.3 K using a Micromeritics TriStar II 3020 surface area and porosity analyzer. The specific surface area was calculated using the Brunauer–Emmett–Teller (BET) method, and the pore size distribution was derived from the desorption branch of the isotherms via the Barrett–Joyner–Halenda (BJH) model.

### 4.5 Clinical Samples Collection and Preparation

Serum samples were obtained from the Second Affiliated Hospital of Nanchang University. A total of 776 samples were collected, including patients with BA, COPD, ILD, LCa, and health control (HC). The collection procedure was approved by the Ethics Committee of Biomedical Research of Hospital (Approval No: IIT‐O‐2025‐119) and complied with all relevant ethical regulations. All samples were annotated with age and gender. The higher proportion of male patients in the COPD and LCa groups is consistent with epidemiological data. Fasting peripheral venous blood samples were collected using standard yellow‐top vacutainer tubes containing separation gel, allowed to clot at room temperature, and centrifuged at 3000 rpm for 10 min to separate serum. The supernatant (200 µL) was then mixed with 600 µL of acetonitrile, vortexed, and stored at ‐80°C until analysis. All samples were processed within 3 h of collection.

### 4.6 LDI‐MS Analysis

Matrix materials (ZIF‐67, Co_3_O_4_, TiO_2_, and MTi) were dispersed in DI water at a concentration of 2 mg mL^−^^1^. Standard metabolites (Ser, Phe, Trp, Val, Lys, Lac) were dissolved in DI water at 5 mm. Salt and protein tolerance were assessed by detecting the standard mixture in the presence of NaCl (1 m) or BSA (5 mg mL^−^^1^). For a typical LDI‐MS assay, 1 µL of the matrix suspension was mixed with 1 µL of the analyte solution and spotted onto a stainless‐steel target plate, followed by air drying. MS data were acquired using an Ultraflextreme MALDI‐TOF mass spectrometer (Bruker Daltonics) equipped with a 355 nm Nd:YAG laser. For each sample spot, mass spectra were acquired in positive reflector ion mode by accumulating 1000 laser shots at random positions across the entire spot to mitigate potential signal fluctuations caused by matrix spatial heterogeneity. The laser focus size was set to the default small setting, and 70% laser energy was provided by the FlexControl software. The instrument was pre‐calibrated using α‐cyano‐4‐hydroxycinnamic acid (CHCA) and 2,5‐dihydroxybenzoic acid (DHB) matrices, with the [M+H]^+^ and [2M+H]^+^ monoisotopic peaks of these molecules as calibration standards. For metabolite identification, a 15 T solariX Fourier‐transform ion cyclotron resonance (FT‐ICR) MS (Bruker Daltonics) was used. The instrument was calibrated with sodium formate cluster ions prior to analysis. Data were acquired at 70% laser power with a “medium” laser spot size and a 100 Hz pulse frequency, accumulating 200 shots per spectrum. Raw data were processed using DataAnalysis software (Bruker Daltonics). Putative metabolite identification was performed by matching the accurate m/z values against the Human Metabolome Database (HMDB, https://hmdb.ca/) with a mass tolerance of 2 ppm.

### 4.7 Statistical Analysis and Machine Learning

For each serum sample, three technical replicate spectra were acquired and processed without smoothing. Peak extraction was performed using flexAnalysis software (Bruker Daltonics). Subsequent data pre‐processing, including spectral alignment, total ion current normalization, and standardization, was executed using a custom Python script (version 3.9). Outliers within the dataset were evaluated and removed using the Interquartile Range (IQR) method (1.5 × IQR). The identification of the metabolites was conducted on the Human Metabolome Database (HMDB, http://www.hmdb.ca/) based on the accurate *m/z* value. Machine learning models, including Neural Networks (NN), Support Vector Machines (SVM), K‐Nearest Neighbors (KNN), and Naïve Bayes (NB), were implemented using the *Orange* package in Python. Feature selection was performed using built‐in scoring methods (Information Gain, Gain Ratio, Gini, ReliefF, and FCBF). The reliability of the model was assessed using ROC curves. Classifiers were evaluated using stratified 5‐fold cross‐validation and leave‐one‐out cross‐validation. Metrices such as accuracy, precision, sensitivity, recall, and F1‐scores were calculated based on true positive (TP), false positive (FP), true negative (TN), and false negative (FN) values. Heatmaps and violin plots were generated using OmicStudio (https://www.omicstudio.cn), and metabolic pathway analysis was conducted with MetaboAnalyst 6.0 (https://www.metaboanalyst.ca).

All formal statistical analyses were performed using GraphPad Prism (8.0.2). The clinical cohort included 776 independent biological samples (n = 210 for HC, n = 101 for BA, n = 109 for COPD, n = 172 for ILD, and n = 184 for LCa). Prior to statistical testing, the normality of the data distribution and homogeneity of variances were assessed. To prevent selection bias and maintain real‐world clinical representativeness, the natural epidemiological demographic distributions (including gender disparities in COPD and LCa) were preserved without artificial resampling. To avoid random technical noise, three independent technical replicates per biological sample were tested and used in the workflow. For each classification task, the data matrix was randomly partitioned into an 80% discovery set and a validation set, the 20% validation set was strictly kept unseen during model training and hyperparameter optimization to ensure an unbiased evaluation of the model's performance. Evaluation metrics insensitive to class imbalance, such as AUC and confusion matrices, were utilized to ensure unbiased model assessment. For variables meeting these assumptions, a two‐tailed Student's *t*‐test was used for two‐group comparisons. For multi‐group comparisons, a One‐way Analysis of Variance (ANOVA) followed by Tukey's post‐hoc test was applied. Non‐parametric alternatives (e.g., Mann–Whitney U test or Kruskal–Wallis test) were utilized when data did not follow a normal distribution. The overall testing level (alpha) was set at 0.05, and p‐values were strictly adjusted using the False Discovery Rate (FDR) method. Significance levels were designated as ^*^ *p* < 0.05, ^**^ *p* < 0.01, ^***^ *p* < 0.001, and ^****^ *p* < 0.0001. Unless stated otherwise, all quantitative data are presented as the mean ± standard deviation (SD) from at least three independent experimental or technical replicates.

## Author Contributions

J. Chen initiated the project. J. Chen, C. Wang, and X. Yu conceived and conducted the experiments. J. Chen and C. Wang analyzed the data. Y. Jiang, Y. Qu, H. Fu, and Y. Bu assisted with MS analysis. J. Chen, X. Zhang, and Z. Nie co‐wrote the paper. All authors contributed to the result discussions and manuscript review.

## Conflicts of Interest

The authors declare no conflicts of interest.

## Supporting information




**Supporting File**: advs75597‐sup‐0001‐SuppMat.docx.

## Data Availability

Hollow Co_3_O_4_/TiO_2_ heterojunctions are engineered for high‐throughput laser desorption/ionization mass spectrometry. The built‐in electric field drives efficient charge separation and photothermal conversion, while the hierarchical mesoporous network enables robust salt and protein tolerance. This synergistic platform achieves direct, highly sensitive metabolic phenotyping of complex biofluids, promising significant advances in precision clinical diagnostics
