# Supplementary material for: Heterojunction‐Engineered Mass Spectrometry Platform for Deciphering Serum Metabolic Fingerprints in Diagnosis of Respiratory Diseases
Source: Adv Sci (Weinh). 2026 May 8;13(43):e75597. doi: 10.1002/advs.75597 (PMC13335958; doi:10.1002/advs.75597)
Supplement: Supplementary file 1 — Supporting File: advs75597‐sup‐0001‐SuppMat.docx. [file ADVS-13-e75597-s001.docx]

Heterojunction-Engineered Mass Spectrometry Platform for Deciphering Serum Metabolic Fingerprints in Diagnosis of Respiratory Diseases

Junyu Chen^1,2^*, Chaoqi Wang^1^, Xi Yu^2^, Yuming Jiang^3^, Yijiao Qu^2^, Huihui Fu^1^, Yuyin Bu^1^, Xiaoyong Zhang^4^*, Zongxiu Nie^2,5^*

1 Jiangxi Province Key Laboratory of Immunology and Inflammation, Jiangxi Provincial Clinical Research Center for Laboratory Medicine, Department of Clinical Laboratory, The Second Affiliated Hospital, Jiangxi Medical College, Nanchang University, Nanchang, 330006, China.

2 Beijing National Laboratory for Molecular Sciences, Key Laboratory of Analytical Chemistry for Living Biosystems, Institute of Chemistry, Chinese Academy of Sciences, Beijing, 100190, China.

3 Department of Computational Biomedicine, Smidt Heart Institute, and Advanced Clinical Biosystems Research Institute, Cedars Sinai Medical Center, Los Angeles, California 90048, United States.

4 Department of Chemistry, Nanchang University, 999 Xuefu Avenue, Nanchang, 330031, China.

5 State Key Laboratory of Tropic Ocean Engineering Materials and Materials Evaluation, School of Marine Science and Engineering, Hainan University, Haikou 570228, China.


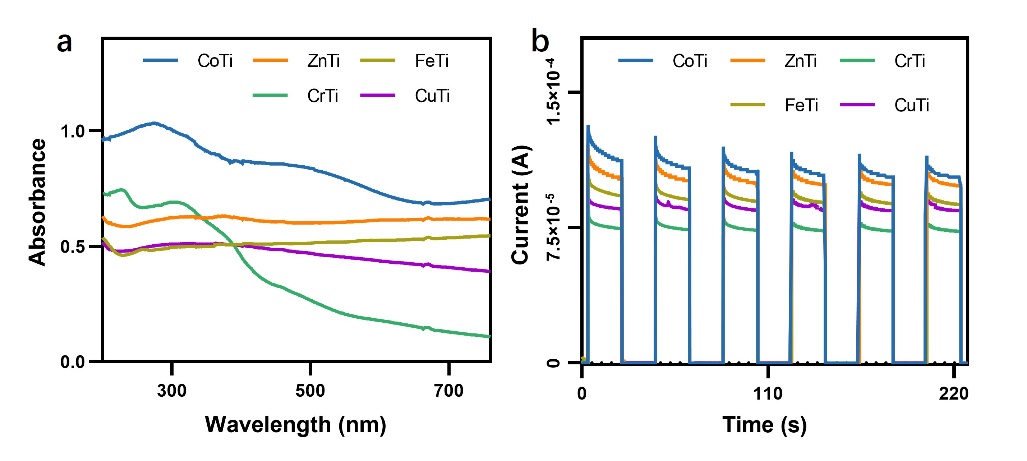


Figure S1. (a) UV-Vis absorption spectra and (b) Transient photocurrent responses of the five MTi heterojunctions.


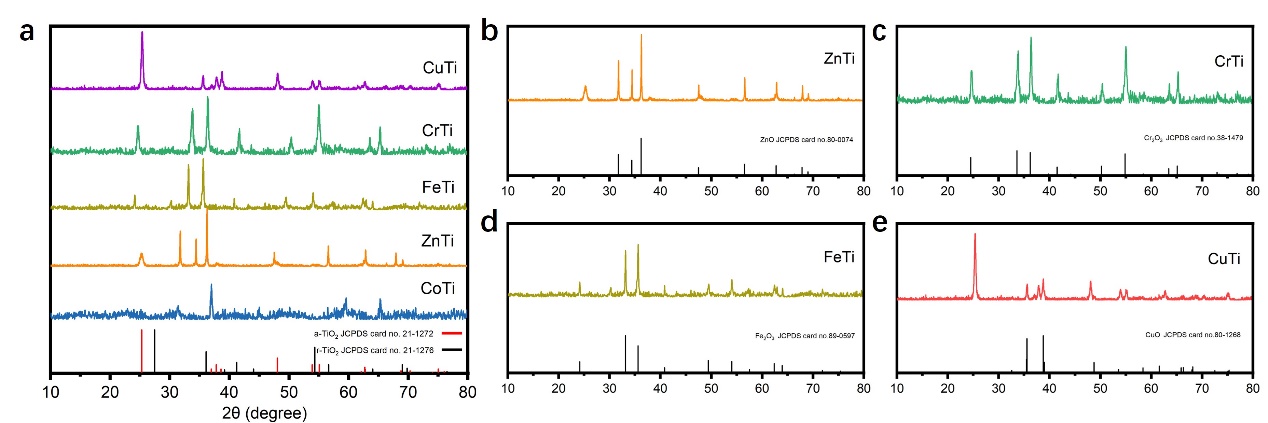


Figure S2. XRD patterns of (a) all MTi composites, (b) ZnTi, (c) CrTi, (d) FeTi, and (e) CuTi, with reference patterns for the respective metal oxides and TiO₂.


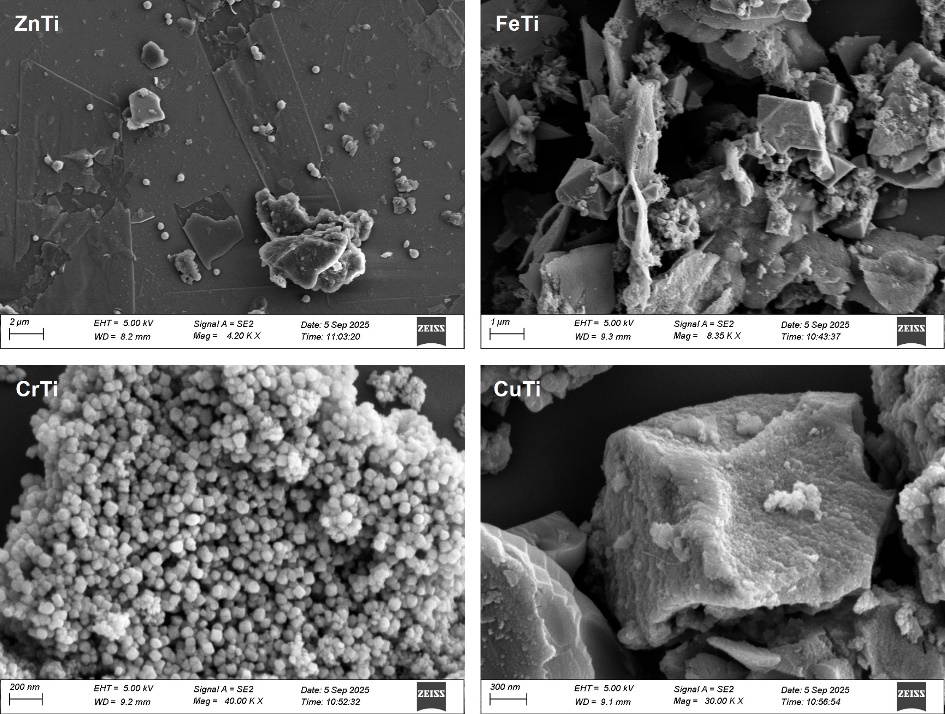


Figure S3. SEM images of ZnTi, FeTi, CrTi, and CuTi.


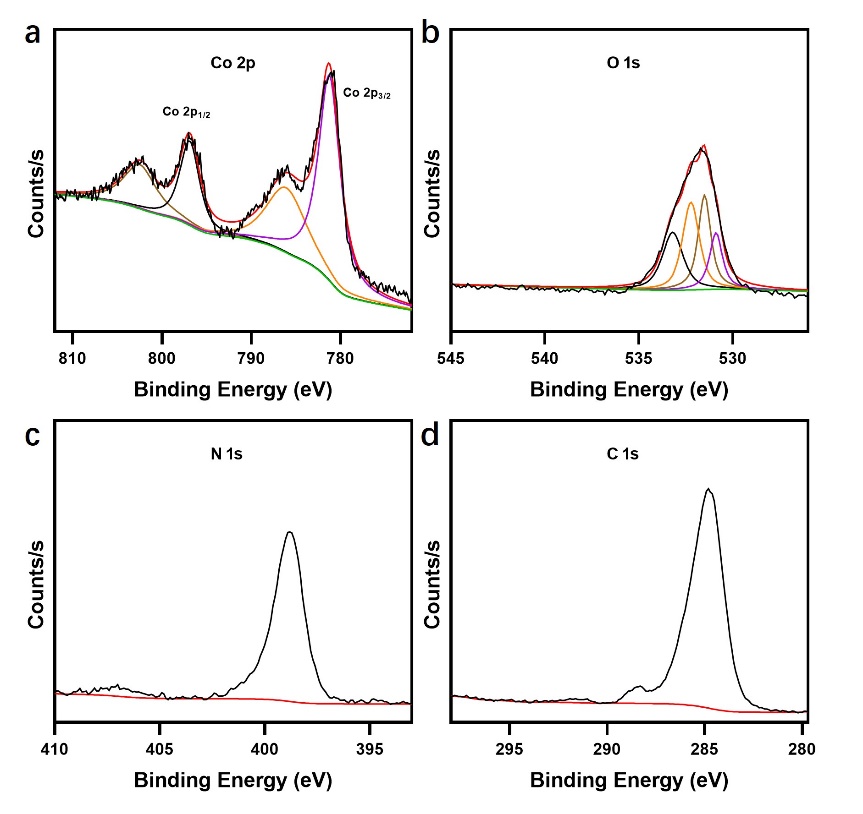


Figure S4. High-resolution XPS spectra of ZIF-67: (a) Co 2p, (b) O 1s, (c) N 1s, and (d) C 1s.


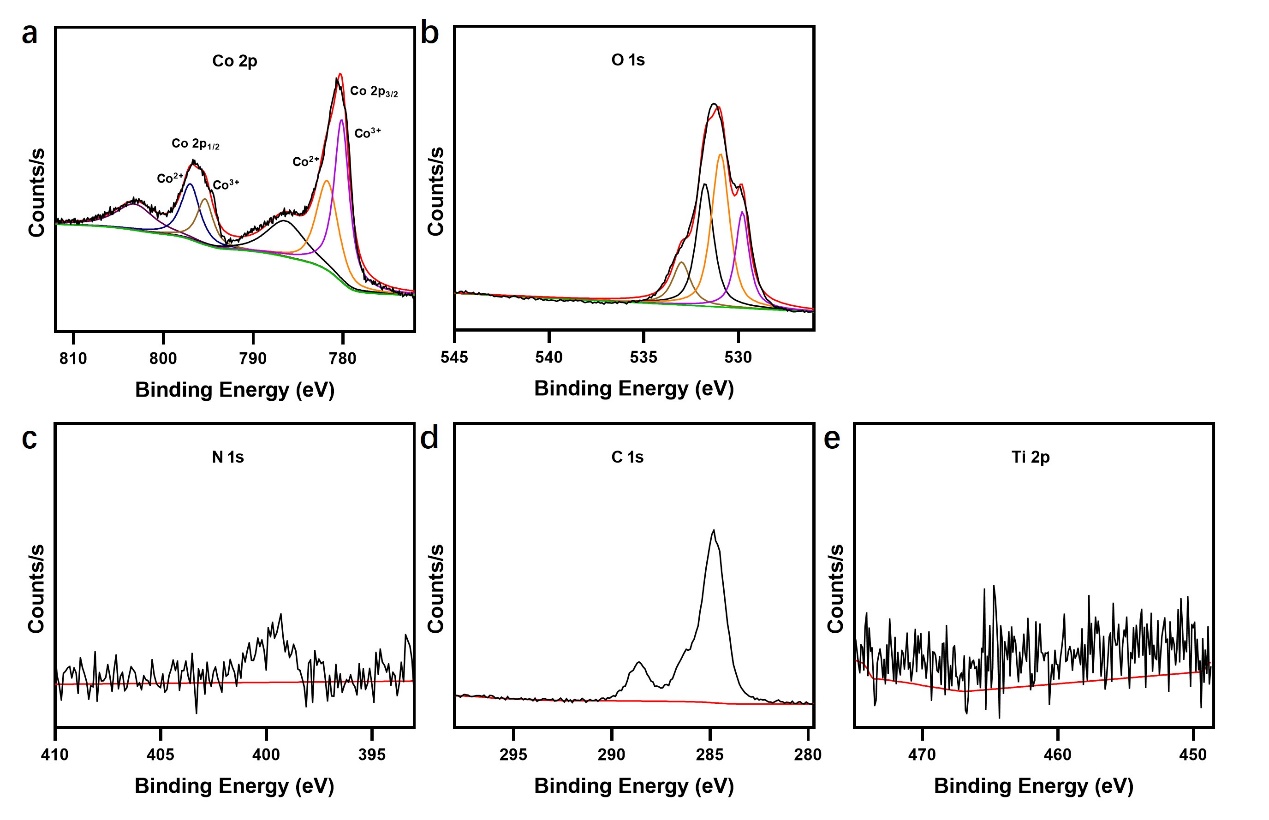


Figure S5. High-resolution XPS spectra of Co₃O₄: (a) Co 2p, (b) O 1s, (c) N 1s, (d) C 1s, and (e) Ti 2p.


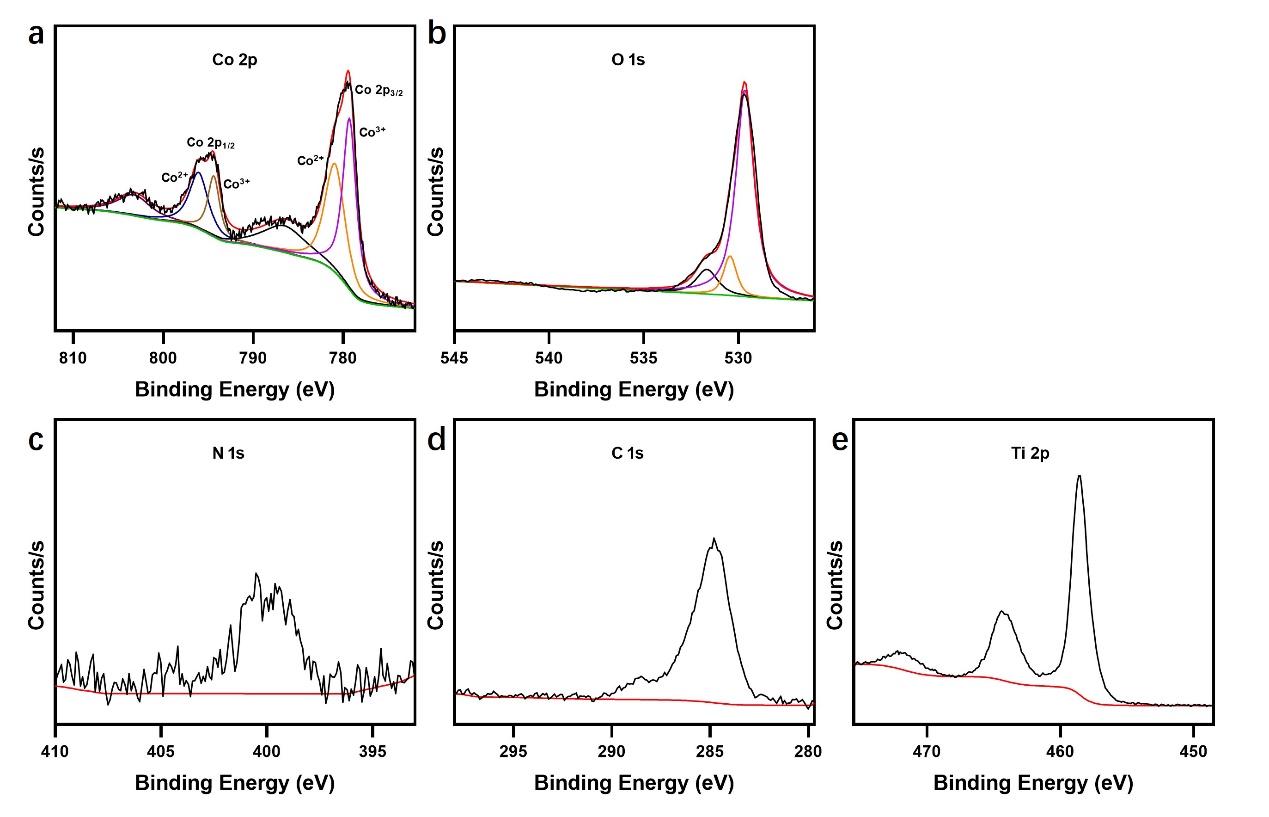


Figure S6. High-resolution XPS spectra of CoTi: (a) Co 2p, (b) O 1s, (c) N 1s, (d) C 1s, and (e) Ti 2p.


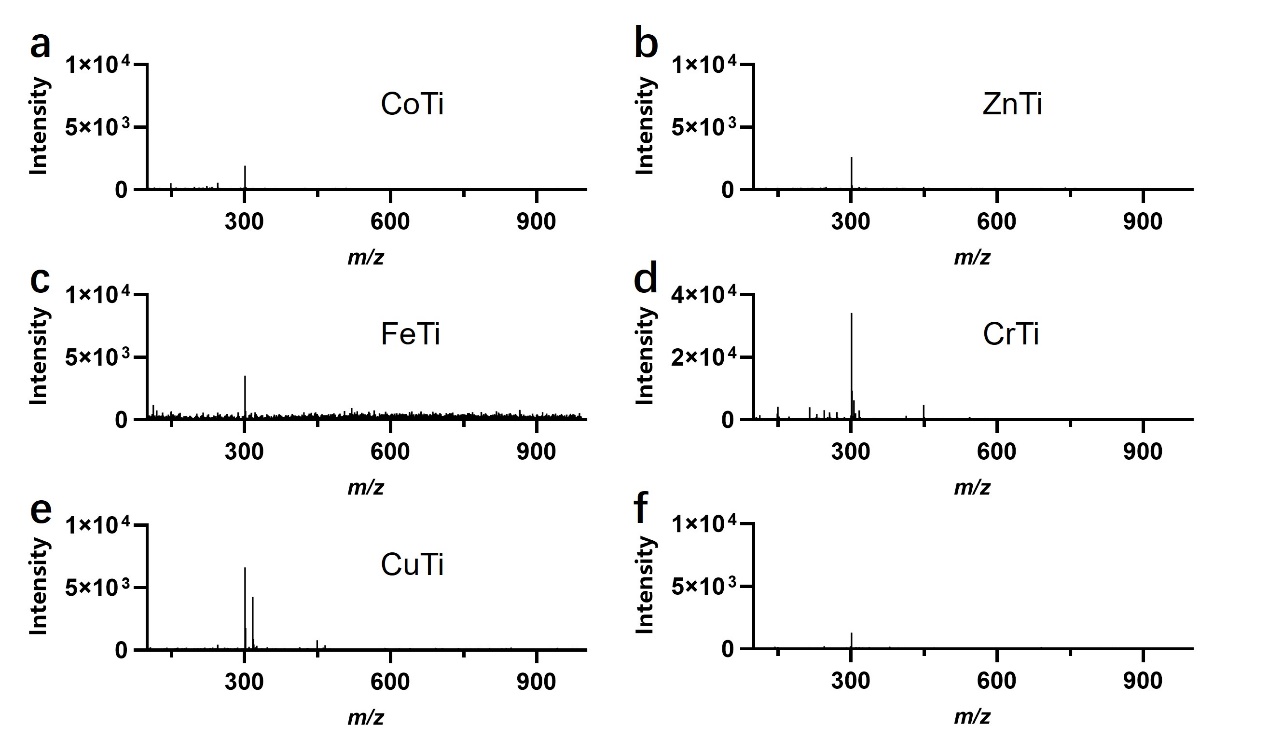


Figure S7. LDI-MS background signals of (a-e) the five MTi matrix and (f) the blank target plate.


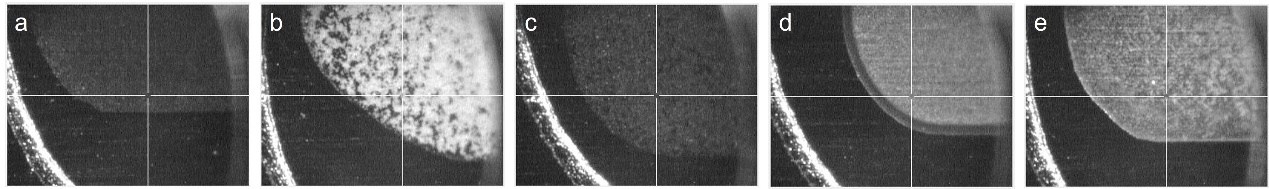


Figure S8. Optical images of dried matrix-analyte spots for (a) CoTi, (b) ZnTi, (c) FeTi, (d) CrTi, and (e) CuTi.


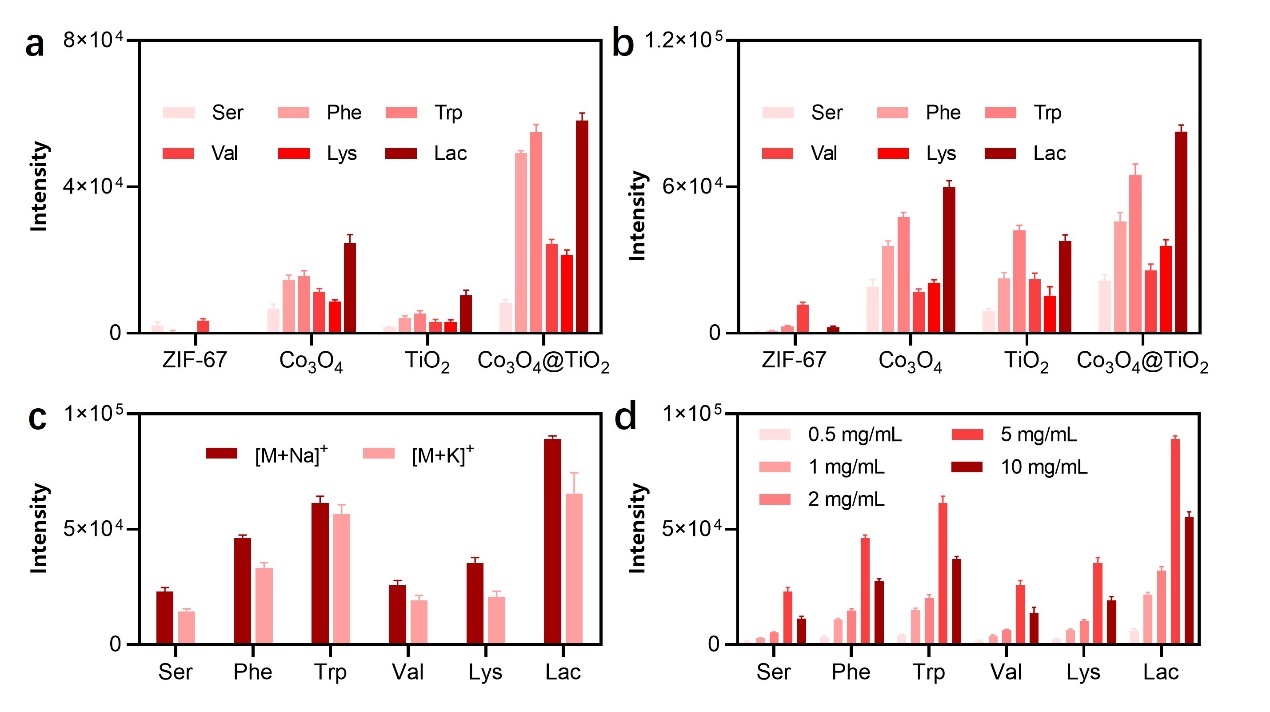


Figure S9. Performance comparison of CoTi and its precursors: Signal intensities in (a) 1 M NaCl and (b) 5 mg mL⁻¹ BSA. (c) Ratio of [M+Na]⁺ to [M+K]⁺ adduct signals for standard metabolites using CoTi. (d) Effect of CoTi matrix concentration on signal intensity.


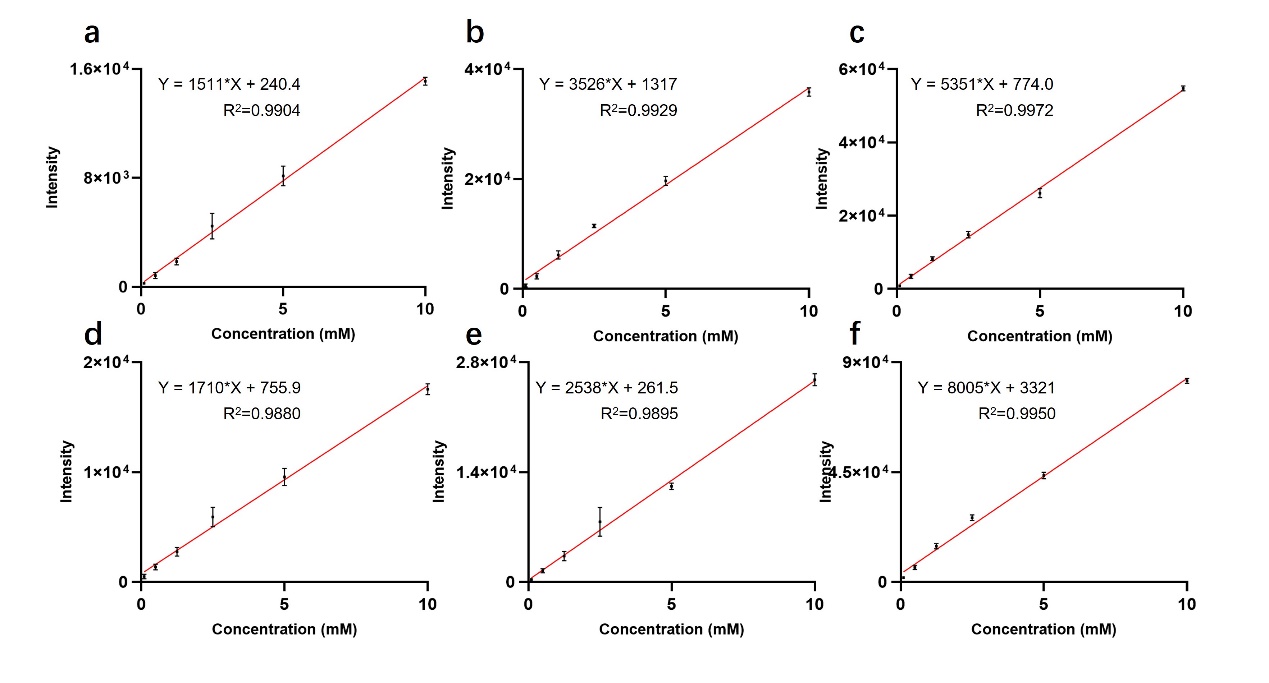


Figure S10. Linear calibration curves for the six standard metabolites: (a) Ser, (b) Phe, (c) Trp, (d) Val, (e) Lys, and (f) Lac.


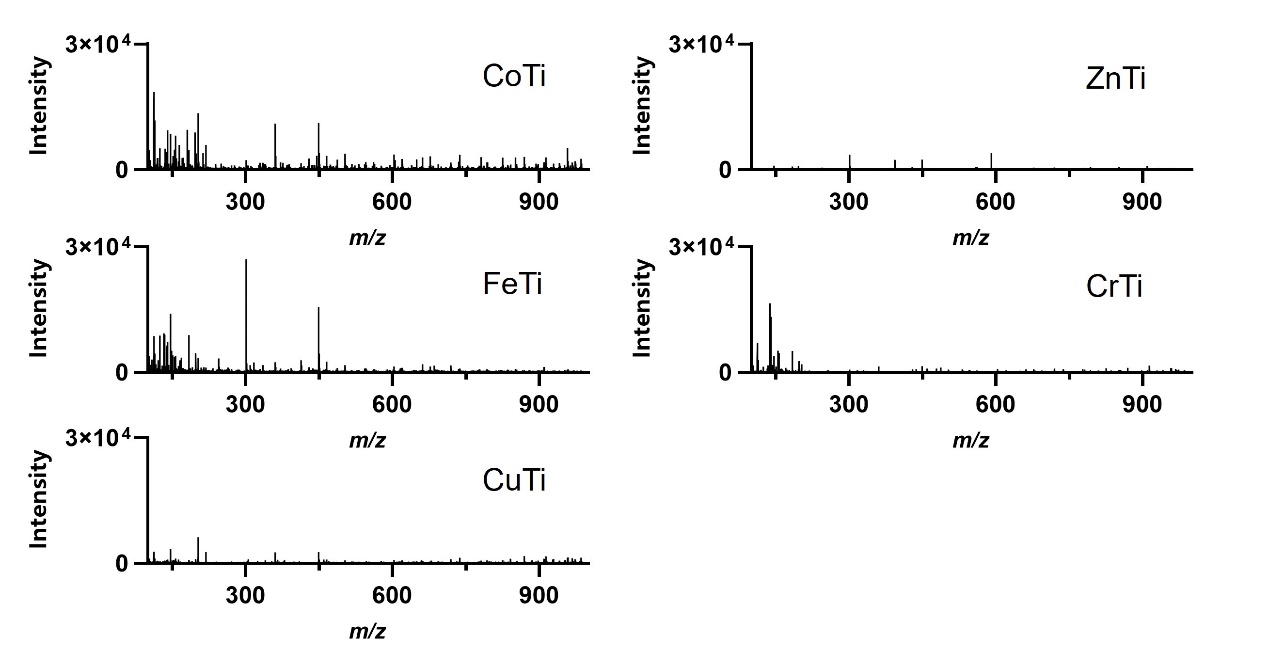


Figure S11. Representative serum metabolic fingerprints obtained using CoTi, ZnTi, FeTi, CrTi, and CuTi matrix.


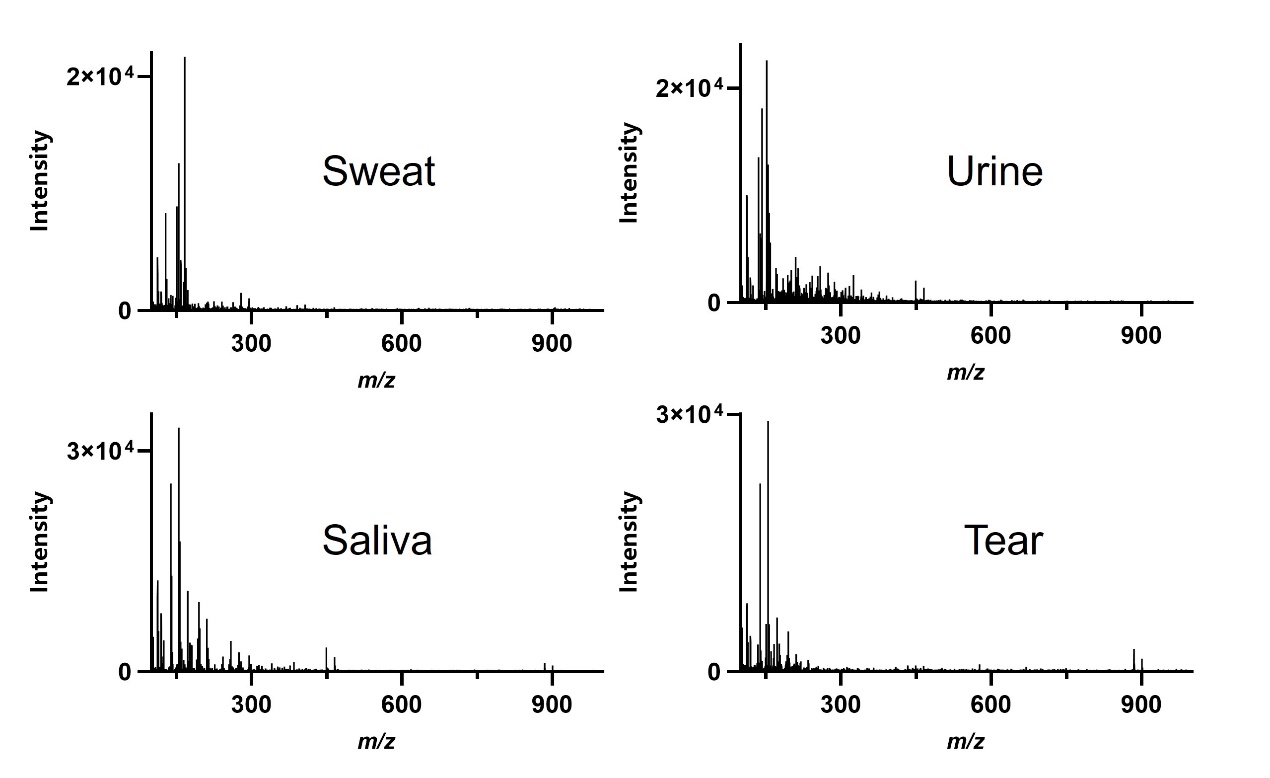


Figure S12. Metabolic fingerprinting of various biofluids (sweat, urine, saliva, tears) using the CoTi matrix.


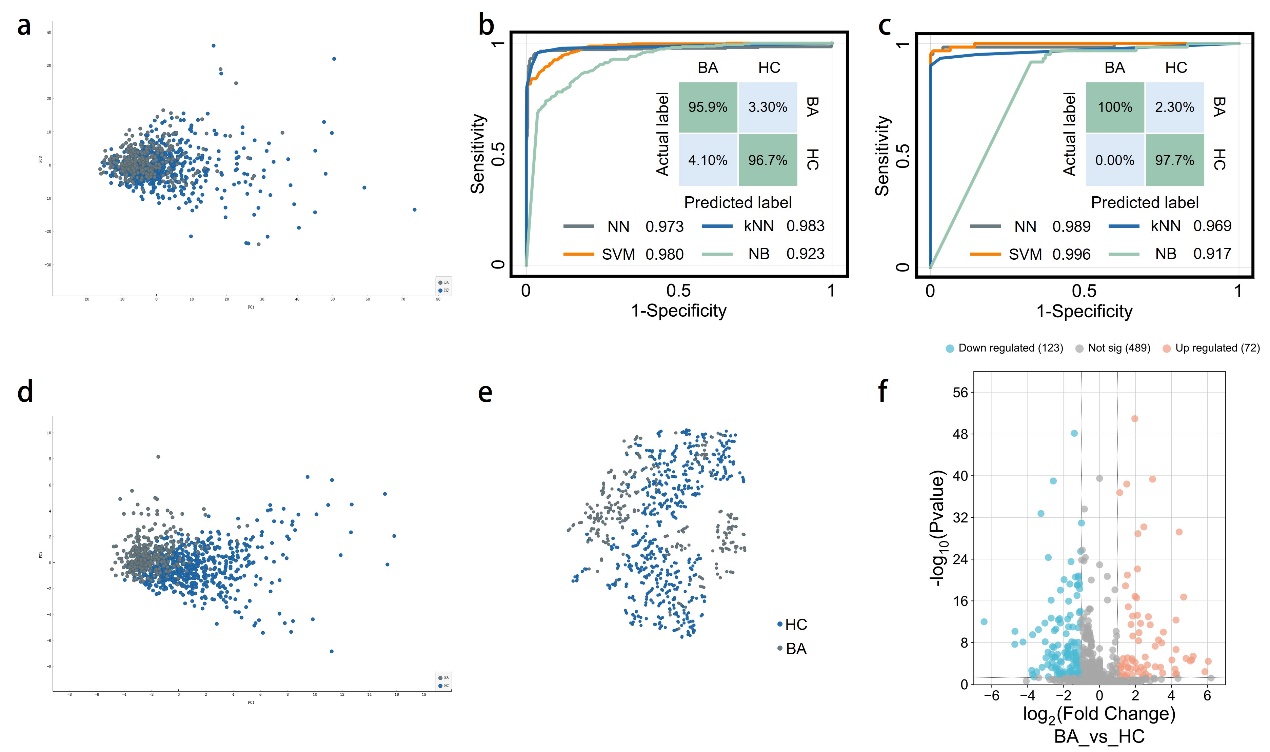


Figure S13. Discrimination of BA from HC using the full metabolic spectrum: (a) PCA plot, (b) ROC curves (discovery set), (c) ROC curves (validation set), (d) PCA plot with top 10 features, (e) t-SNE plot, and (f) Volcano plot of differential m/z features.


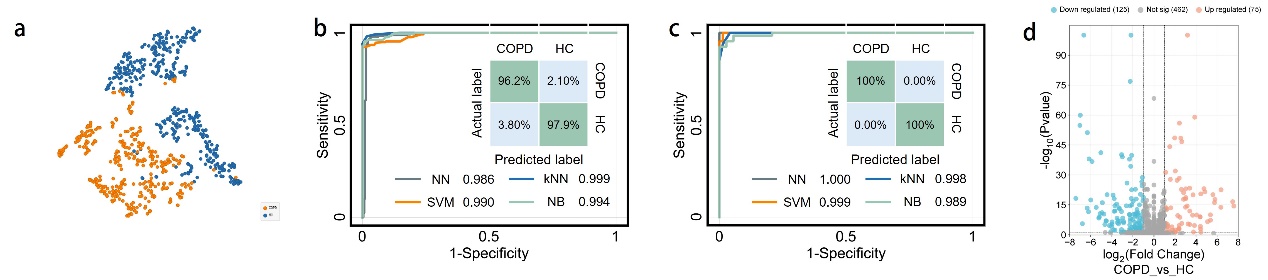


Figure S14. Discrimination of COPD from HC using the full metabolic spectrum: (a) t-SNE plot, (b) ROC curves (discovery set), (c) ROC curves (validation set), and (d) Volcano plot.


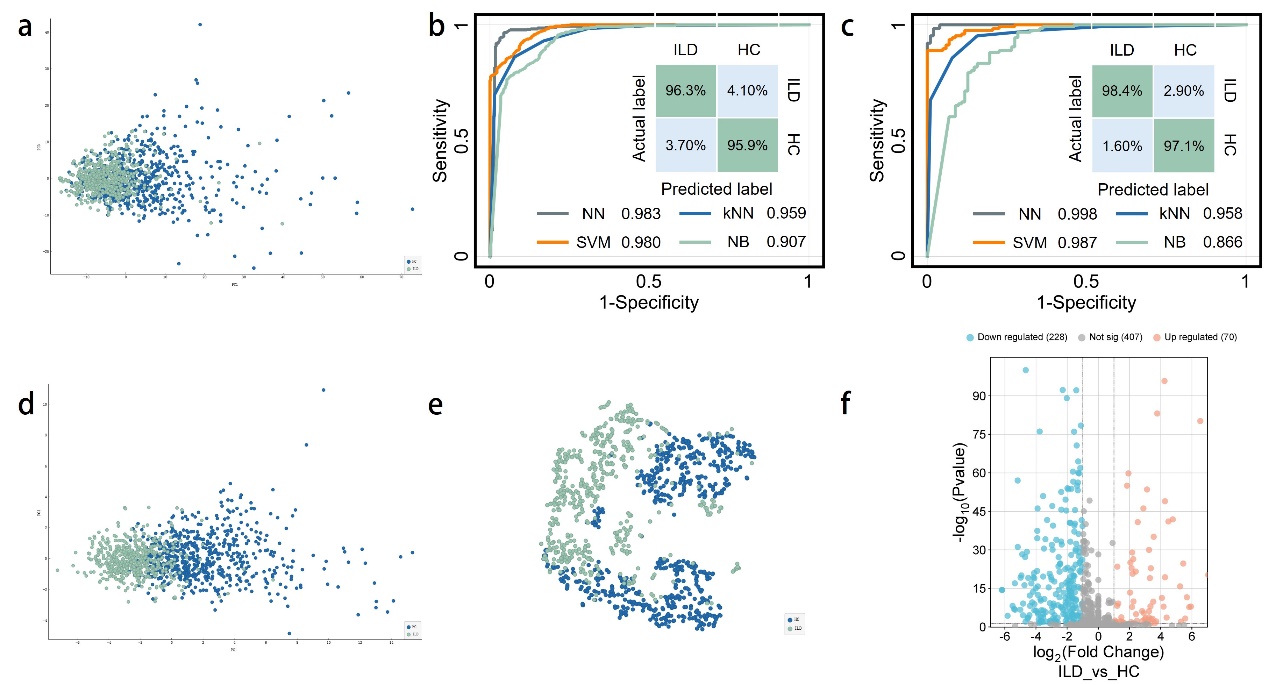


Figure S15. Discrimination of ILD from HC using the full metabolic spectrum: (a) PCA plot, (b) ROC curves (discovery set), (c) ROC curves (validation set), (d) PCA plot with top 10 features, (e) t-SNE plot, and (f) Volcano plot.


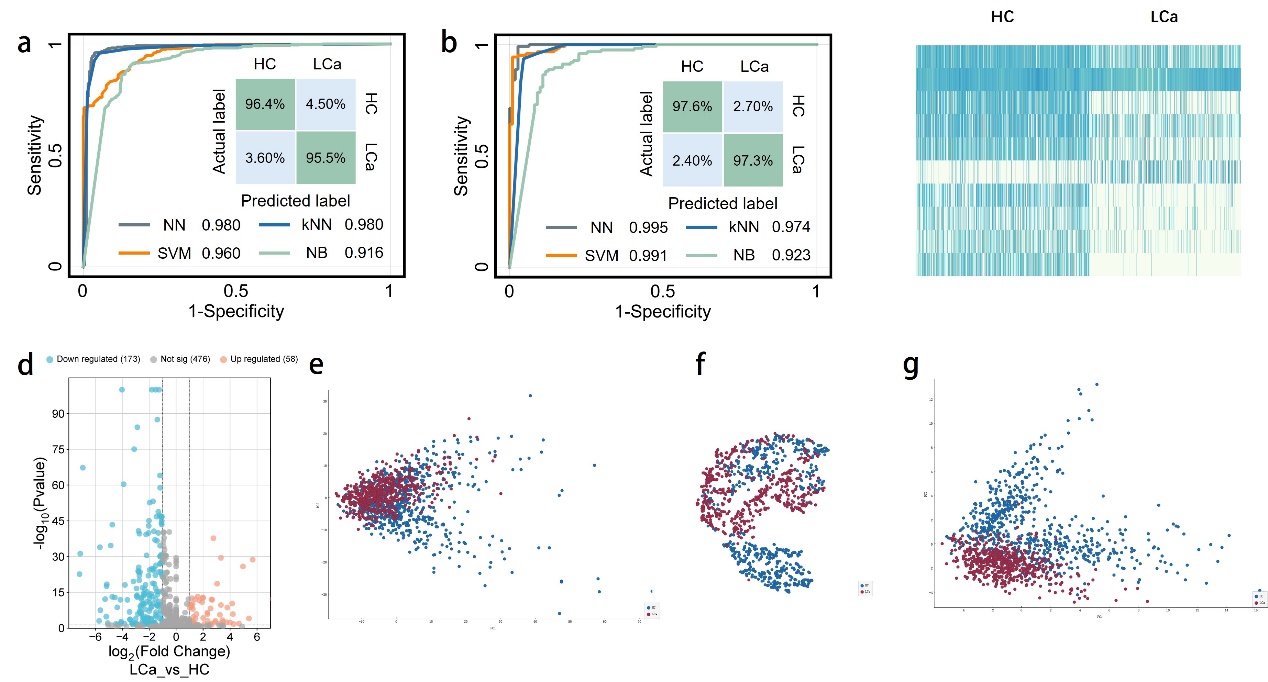


Figure S16. Discrimination of LCa from HC: (a, b) ROC curves using the full spectrum in discovery and validation sets, (c) Heatmap of top 10 features, (d) Volcano plot, (e) PCA plot (full spectrum), (f) t-SNE plot (full spectrum), and (g) PCA plot using the top 10 feature panel.


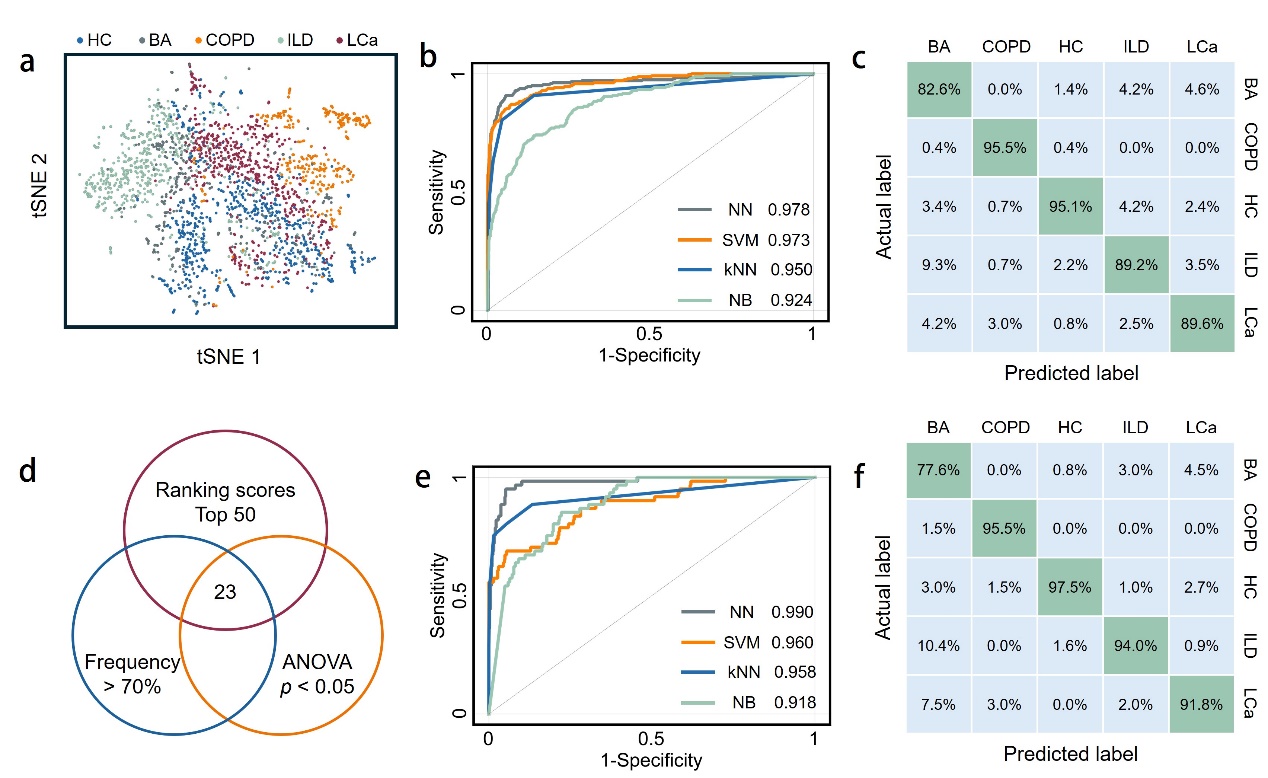


Figure S17. Five-group classification using the full metabolic spectrum: (a) t-SNE plot, (b) ROC curves (discovery set), (c) Confusion matrix (discovery set), (d) Venn diagram for feature selection, (e) ROC curves (validation set), and (f) Confusion matrix (validation set).


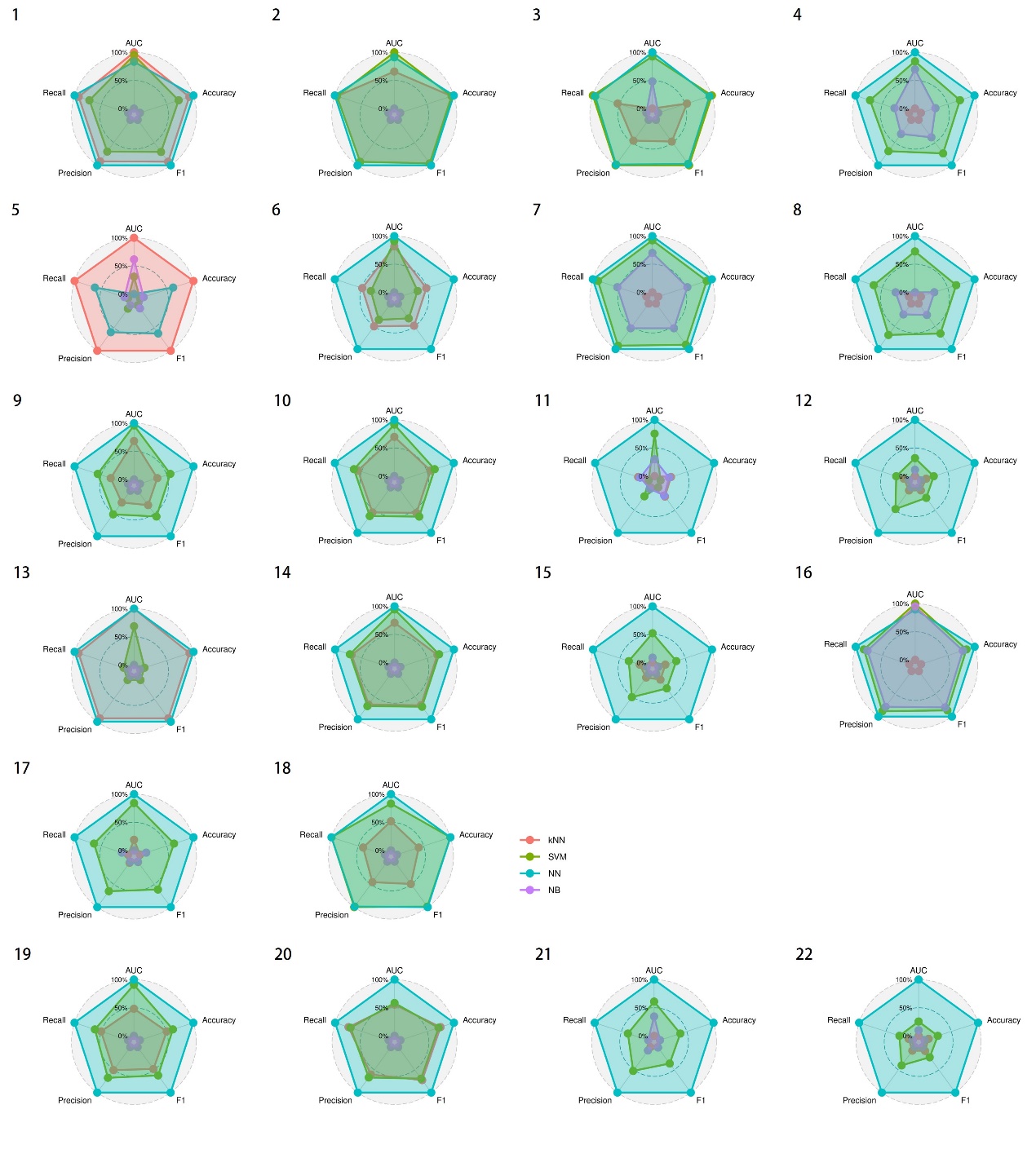


Figure S18. Radar chart comparing the performance (AUC) of NN, SVM, KNN, and NB models across all classification tasks.


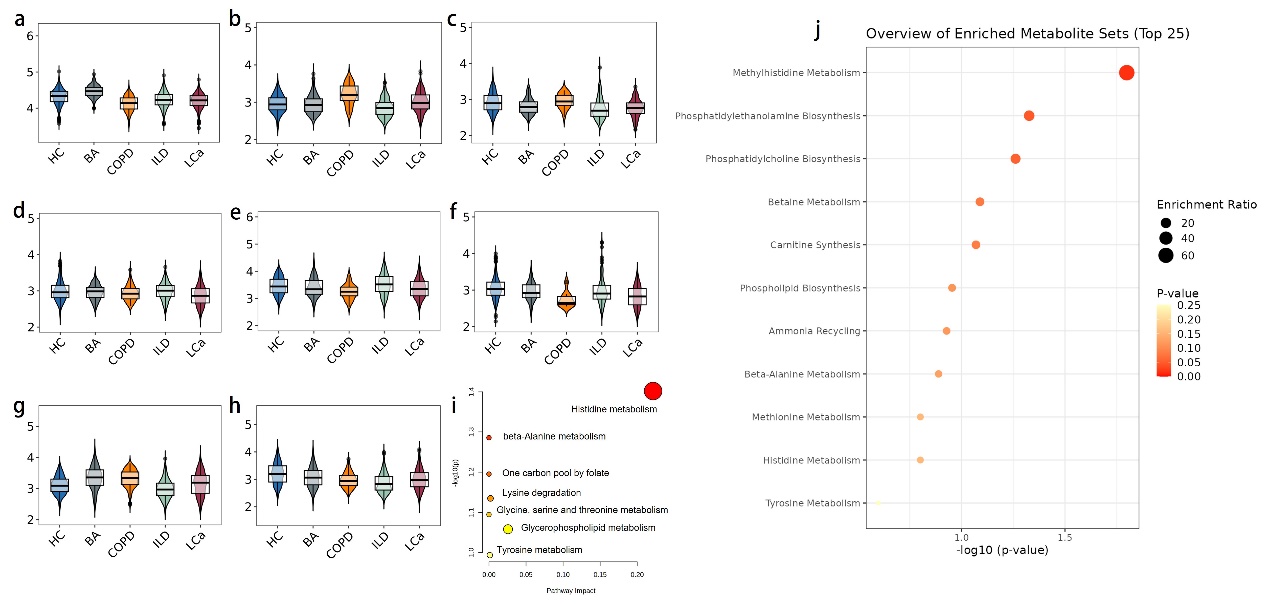


Figure S19. Relative abundance of eight potential biomarkers: (a) Histidine, (b) Hydroxydodecanedioic acid, (c) Lysylglutamine, (d) Trimethylammoniobutanoic acid, (e) Dihydroxycinnamic acid glucuronide, (f) Hexadecanedioic acid, (g) Vanillylmandelic acid, and (h) Creatinine across the five groups. (i) Pathway enrichment analysis and (j) Overview of the top 25 enriched metabolite sets based on the identified metabolites.


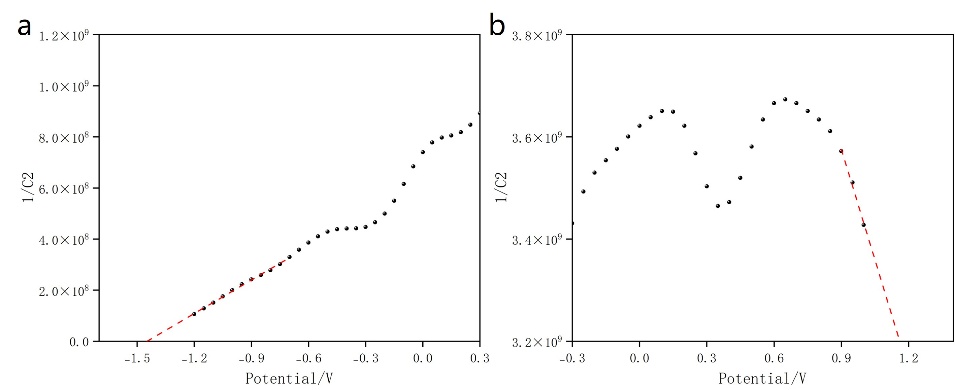


Figure S20. Mott-Schottky plots of the synthesized (a) TiO_2_ and (b) Co_3_O_4_ precursors, measured at a constant frequency of 2 kHz. The distinct positive slope observed in panel (a) indicates the typical n-type semiconducting characteristic of TiO_2_. Conversely, the pronounced negative slope in the high-potential region of panel (b) confirms the p-type semiconducting nature of Co_3_O_4_. The red dashed lines illustrate the linear extrapolations of the space-charge capacitance regions used for semiconductor type determination.


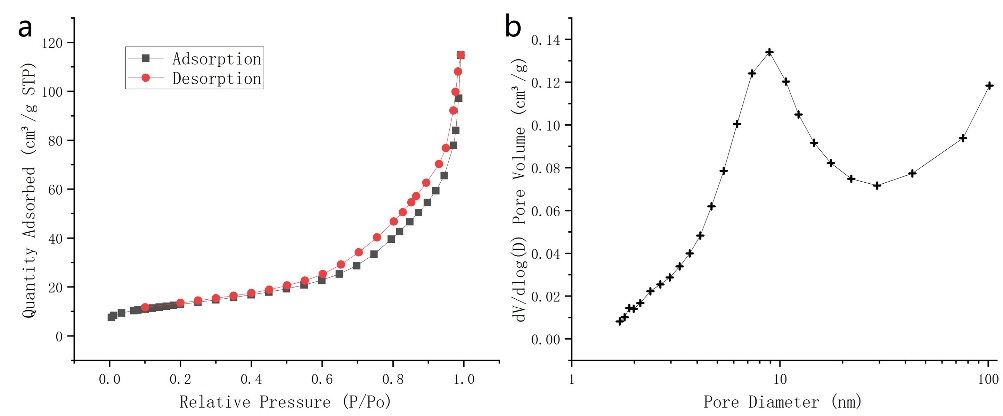


Figure S21. (a) Nitrogen adsorption–desorption isotherms and (b) BJH pore size distribution of the CoTi nano-matrix.

Table S1. Demographic Information of the Study Cohort.

| **Group** | **Number** | **Gender** | | **Age** |
| --- | --- | --- | --- | --- |
|  |  | **Male** | **Female** |  |
| HC | 210 | 107 | 103 | 60 (17-95) |
| BA | 101 | 46 | 55 | 66 (14-93) |
| COPD | 109 | 95 | 14 | 68 (43-92) |
| ILD | 172 | 84 | 88 | 65 (27-87) |
| LCa | 184 | 140 | 44 | 65 (35-89) |

Table S2. Performance of Classifiers for Discriminating BA from HC (Discovery Set, Full Spectrum).

| **Models** | **AUC** | **Accuracy** | **F1** | **Precision** | **Recall** |
| --- | --- | --- | --- | --- | --- |
| kNN | 0.983 | 0.955 | 0.955 | 0.955 | 0.955 |
| SVM | 0.980 | 0.929 | 0.928 | 0.930 | 0.929 |
| NN | 0.973 | 0.965 | 0.965 | 0.965 | 0.965 |
| NB | 0.923 | 0.835 | 0.839 | 0.849 | 0.835 |

Table S3. Performance of Classifiers for Discriminating BA from HC (Validation Set, Full Spectrum).

| **Models** | **AUC** | **Accuracy** | **F1** | **Precision** | **Recall** |
| --- | --- | --- | --- | --- | --- |
| kNN | 0.969 | 0.968 | 0.967 | 0.969 | 0.968 |
| SVM | 0.996 | 0.968 | 0.967 | 0.969 | 0.968 |
| NN | 0.989 | 0.984 | 0.984 | 0.984 | 0.984 |
| NB | 0.917 | 0.581 | 0.573 | 0.791 | 0.581 |

Table S4. Top 10 Key m/z Features for BA vs. HC Classification.

| ***m/z* feature** | **Info. gain** | **Gain ratio** | **Gini** | **ReliefF** | **FCBF** | ***p*-value** | ***p*-value (FDR adj)** |
| --- | --- | --- | --- | --- | --- | --- | --- |
| 231.05 | 0.339 | 0.206 | 0.155 | 0.068 | 3.604E-01 | 9.394E-70 | 9.394E-69 |
| 365.11 | 0.259 | 0.132 | 0.142 | 0.061 | 2.201E-01 | 5.914E-64 | 2.957E-63 |
| 221.04 | 0.213 | 0.112 | 0.123 | 0.038 | 1.781E-01 | 1.592E-29 | 2.654E-29 |
| 233.08 | 0.204 | 0.123 | 0.107 | 0.065 | 1.882E-05 | 1.062E-54 | 3.539E-54 |
| 325.18 | 0.154 | 0.079 | 0.092 | 0.040 | 1.213E-05 | 1.325E-29 | 2.651E-29 |
| 303.14 | 0.153 | 0.078 | 0.091 | 0.076 | 1.194E-05 | 8.070E-20 | 1.009E-19 |
| 173.06 | 0.148 | 0.094 | 0.075 | 0.015 | 1.348E-01 | 2.767E-42 | 6.918E-42 |
| 301.14 | 0.122 | 0.061 | 0.073 | 0.028 | 9.167E-06 | 2.586E-29 | 3.694E-29 |
| 136.05 | 0.086 | 0.043 | 0.054 | 0.025 | 6.301E-06 | 5.498E-15 | 6.109E-15 |
| 184.07 | 0.074 | 0.046 | 0.045 | 0.011 | 6.239E-06 | 5.701E-07 | 5.701E-07 |

Table S5. Performance of Classifiers for Discriminating BA from HC (Discovery Set, Feature Panel).

| **Models** | **AUC** | **Accuracy** | **F1** | **Precision** | **Recall** |
| --- | --- | --- | --- | --- | --- |
| kNN | 0.900 | 0.868 | 0.865 | 0.867 | 0.868 |
| SVM | 0.950 | 0.900 | 0.900 | 0.900 | 0.900 |
| NN | 0.954 | 0.897 | 0.898 | 0.899 | 0.897 |
| NB | 0.926 | 0.831 | 0.833 | 0.839 | 0.831 |

Table S6. Performance of Classifiers for Discriminating BA from HC (Validation Set, Feature Panel).

| **Models** | **AUC** | **Accuracy** | **F1** | **Precision** | **Recall** |
| --- | --- | --- | --- | --- | --- |
| kNN | 0.909 | 0.855 | 0.847 | 0.858 | 0.855 |
| SVM | 0.956 | 0.898 | 0.895 | 0.898 | 0.898 |
| NN | 0.965 | 0.914 | 0.912 | 0.916 | 0.914 |
| NB | 0.948 | 0.871 | 0.872 | 0.876 | 0.871 |

Table S7. Demographic Information of the Matched COPD and HC Sub-cohort.

| **Group** | **Number** | **Gender** | | **Age** |
| --- | --- | --- | --- | --- |
|  |  | **Male** | **Female** |  |
| HC | 122 | 107 | 15 | 61 (17-95) |
| COPD | 109 | 95 | 14 | 68 (43-92) |

Table S8. Performance of Classifiers for Discriminating COPD from HC (Discovery Set, Full Spectrum).

| **Models** | **AUC** | **Accuracy** | **F1** | **Precision** | **Recall** |
| --- | --- | --- | --- | --- | --- |
| kNN | 0.999 | 0.982 | 0.982 | 0.982 | 0.982 |
| SVM | 0.990 | 0.953 | 0.953 | 0.957 | 0.953 |
| NN | 0.986 | 0.971 | 0.971 | 0.971 | 0.971 |
| NB | 0.994 | 0.955 | 0.955 | 0.955 | 0.955 |

Table S9. Performance of Classifiers for Discriminating COPD from HC (Validation Set, Full Spectrum).

| **Models** | **AUC** | **Accuracy** | **F1** | **Precision** | **Recall** |
| --- | --- | --- | --- | --- | --- |
| kNN | 0.998 | 0.978 | 0.978 | 0.979 | 0.978 |
| SVM | 0.999 | 0.971 | 0.971 | 0.973 | 0.971 |
| NN | 1.000 | 1.000 | 1.000 | 1.000 | 1.000 |
| NB | 0.989 | 0.957 | 0.957 | 0.958 | 0.957 |

Table S10. Top 10 Key m/z Features for COPD vs. HC Classification.

| ***m/z* feature** | **Info. gain** | **Gain ratio** | **Gini** | **ReliefF** | **FCBF** | **p-value** | **p-value (FDR adj)** |
| --- | --- | --- | --- | --- | --- | --- | --- |
| 303.14 | 0.566 | 0.342 | 0.314 | 0.097 | 7.432E-01 | 3.046E-53 | 3.046E-52 |
| 325.18 | 0.471 | 0.293 | 0.268 | 0.043 | 5.651E-05 | 1.607E-37 | 3.283E-37 |
| 388.24 | 0.292 | 0.240 | 0.153 | 0.040 | 3.573E-05 | 1.642E-37 | 3.283E-37 |
| 332.25 | 0.285 | 0.207 | 0.161 | 0.033 | 3.155E-05 | 1.145E-41 | 3.815E-41 |
| 457.34 | 0.265 | 0.137 | 0.164 | 0.122 | 2.200E-05 | 4.775E-43 | 2.387E-42 |
| 379.06 | 0.205 | 0.129 | 0.129 | 0.035 | 1.882E-05 | 1.055E-27 | 1.758E-27 |
| 368.21 | 0.184 | 0.216 | 0.098 | 0.026 | 2.484E-05 | 1.520E-12 | 1.520E-12 |
| 194.03 | 0.171 | 0.137 | 0.098 | 0.075 | 1.798E-05 | 4.516E-19 | 5.645E-19 |
| 217.10 | 0.169 | 0.135 | 0.108 | 0.026 | 1.767E-01 | 4.454E-21 | 6.362E-21 |
| 209.99 | 0.152 | 0.120 | 0.086 | 0.049 | 1.552E-05 | 7.587E-13 | 8.430E-13 |

Table S11. Performance of Classifiers for Discriminating COPD from HC (Discovery Set, Feature Panel).

| **Models** | **AUC** | **Accuracy** | **F1** | **Precision** | **Recall** |
| --- | --- | --- | --- | --- | --- |
| kNN | 0.956 | 0.881 | 0.881 | 0.881 | 0.881 |
| SVM | 0.993 | 0.966 | 0.966 | 0.968 | 0.966 |
| NN | 0.996 | 0.975 | 0.975 | 0.975 | 0.975 |
| NB | 0.984 | 0.932 | 0.932 | 0.932 | 0.932 |

Table S12. Performance of Classifiers for Discriminating COPD from HC (Validation Set, Feature Panel).

| **Models** | **AUC** | **Accuracy** | **F1** | **Precision** | **Recall** |
| --- | --- | --- | --- | --- | --- |
| kNN | 0.988 | 0.942 | 0.942 | 0.944 | 0.942 |
| SVM | 0.996 | 0.971 | 0.971 | 0.973 | 0.971 |
| NN | 0.999 | 0.986 | 0.986 | 0.986 | 0.986 |
| NB | 0.988 | 0.953 | 0.953 | 0.954 | 0.953 |

Table S13. Performance of Classifiers for Discriminating ILD from HC (Discovery Set, Full Spectrum).

| **Models** | **AUC** | **Accuracy** | **F1** | **Precision** | **Recall** |
| --- | --- | --- | --- | --- | --- |
| kNN | 0.959 | 0.886 | 0.885 | 0.887 | 0.886 |
| SVM | 0.980 | 0.913 | 0.913 | 0.913 | 0.913 |
| NN | 0.983 | 0.961 | 0.961 | 0.961 | 0.961 |
| NB | 0.907 | 0.850 | 0.850 | 0.861 | 0.850 |

Table S14. Performance of Classifiers for Discriminating ILD from HC (Validation Set, Full Spectrum).

| **Models** | **AUC** | **Accuracy** | **F1** | **Precision** | **Recall** |
| --- | --- | --- | --- | --- | --- |
| kNN | 0.958 | 0.904 | 0.903 | 0.906 | 0.904 |
| SVM | 0.987 | 0.917 | 0.917 | 0.919 | 0.917 |
| NN | 0.998 | 0.978 | 0.978 | 0.978 | 0.978 |
| NB | 0.866 | 0.807 | 0.807 | 0.818 | 0.807 |

Table S15. Top 10 Key m/z Features for ILD vs. HC Classification.

| **m/z feature** | **Info. gain** | **Gain ratio** | **Gini** | **ReliefF** | **FCBF** | **p-value** | **p-value (FDR adj)** |
| --- | --- | --- | --- | --- | --- | --- | --- |
| 301.14 | 0.299 | 0.150 | 0.186 | 0.039 | 2.506E-05 | 3.411E-59 | 1.137E-58 |
| 203.05 | 0.290 | 0.145 | 0.175 | 0.068 | 2.405E-05 | 4.562E-99 | 4.562E-98 |
| 365.11 | 0.275 | 0.147 | 0.172 | 0.046 | 2.379E-05 | 9.097E-58 | 2.274E-57 |
| 233.08 | 0.197 | 0.127 | 0.120 | 0.018 | 1.830E-01 | 1.119E-35 | 1.398E-35 |
| 231.05 | 0.195 | 0.124 | 0.119 | 0.032 | 1.795E-05 | 8.567E-53 | 1.713E-52 |
| 184.07 | 0.193 | 0.106 | 0.126 | 0.049 | 1.593E-01 | 1.289E-24 | 1.432E-24 |
| 136.05 | 0.172 | 0.086 | 0.110 | 0.036 | 1.298E-05 | 1.461E-45 | 2.435E-45 |
| 219.03 | 0.171 | 0.085 | 0.107 | 0.049 | 1.287E-05 | 1.840E-62 | 9.202E-62 |
| 325.18 | 0.154 | 0.083 | 0.101 | 0.013 | 1.212E-05 | 1.765E-09 | 1.765E-09 |
| 104.11 | 0.148 | 0.074 | 0.096 | 0.027 | 1.099E-05 | 8.885E-38 | 1.269E-37 |

Table S16. Performance of Classifiers for Discriminating ILD from HC (Discovery Set, Feature Panel).

| **Models** | **AUC** | **Accuracy** | **F1** | **Precision** | **Recall** |
| --- | --- | --- | --- | --- | --- |
| kNN | 0.897 | 0.823 | 0.822 | 0.823 | 0.823 |
| SVM | 0.940 | 0.807 | 0.805 | 0.836 | 0.807 |
| NN | 0.954 | 0.889 | 0.889 | 0.889 | 0.889 |
| NB | 0.914 | 0.820 | 0.820 | 0.825 | 0.820 |

Table S17. Performance of Classifiers for Discriminating ILD from HC (Validation Set, Feature Panel).

| **Models** | **AUC** | **Accuracy** | **F1** | **Precision** | **Recall** |
| --- | --- | --- | --- | --- | --- |
| kNN | 0.873 | 0.785 | 0.783 | 0.786 | 0.785 |
| SVM | 0.896 | 0.798 | 0.798 | 0.822 | 0.798 |
| NN | 0.945 | 0.868 | 0.868 | 0.868 | 0.868 |
| NB | 0.881 | 0.776 | 0.777 | 0.780 | 0.776 |

Table S18. Demographic Information of the Matched LCa and HC Sub-cohort.

| **Group** | **Number** | **Gender** | | **Age** |  |  | **Stages** |  |  |  |
| --- | --- | --- | --- | --- | --- | --- | --- | --- | --- | --- |
|  |  | **Male** | **Female** |  | T0-1 | T2 | T3 | T4 | Tr | Unknown |
| HC | 142 | 107 | 36 | 62 (29-95) | / | / | / | / | / | / |
| LCa | 182 | 140 | 44 | 65 (35-89) | 29 | 27 | 35 | 37 | 29 | 27 |

Table S19. Performance of Classifiers for Discriminating LCa from HC (Discovery Set, Full Spectrum).

| **Models** | **AUC** | **Accuracy** | **F1** | **Precision** | **Recall** |
| --- | --- | --- | --- | --- | --- |
| kNN | 0.980 | 0.953 | 0.953 | 0.953 | 0.953 |
| SVM | 0.960 | 0.873 | 0.873 | 0.873 | 0.873 |
| NN | 0.980 | 0.960 | 0.960 | 0.960 | 0.960 |
| NB | 0.916 | 0.865 | 0.865 | 0.865 | 0.865 |

Table S20. Performance of Classifiers for Discriminating LCa from HC (Validation Set, Full Spectrum).

| **Models** | **AUC** | **Accuracy** | **F1** | **Precision** | **Recall** |
| --- | --- | --- | --- | --- | --- |
| kNN | 0.974 | 0.937 | 0.937 | 0.937 | 0.937 |
| SVM | 0.991 | 0.941 | 0.941 | 0.941 | 0.941 |
| NN | 0.995 | 0.975 | 0.975 | 0.975 | 0.975 |
| NB | 0.923 | 0.852 | 0.852 | 0.858 | 0.852 |

Table S21. Top 10 Key m/z Features for LCa vs. HC Classification.

| **m/z feature** | **Info. gain** | **Gain ratio** | **Gini** | **ReliefF** | **FCBF** | **p-value** | **p-value (FDR adj)** |
| --- | --- | --- | --- | --- | --- | --- | --- |
| 231.05 | 0.361 | 0.258 | 0.196 | 0.026 | 4.318E-01 | 9.592E-69 | 4.796E-68 |
| 365.11 | 0.312 | 0.168 | 0.184 | 0.036 | 2.799E-05 | 6.370E-77 | 6.370E-76 |
| 332.25 | 0.256 | 0.178 | 0.144 | 0.031 | 2.664E-05 | 3.346E-23 | 3.346E-23 |
| 173.06 | 0.165 | 0.115 | 0.100 | 0.039 | 1.569E-01 | 7.421E-44 | 2.474E-43 |
| 325.18 | 0.158 | 0.087 | 0.102 | 0.019 | 1.263E-05 | 5.651E-43 | 1.130E-42 |
| 301.14 | 0.135 | 0.068 | 0.088 | 0.026 | 9.903E-06 | 1.947E-32 | 2.781E-32 |
| 303.14 | 0.125 | 0.065 | 0.080 | 0.031 | 9.338E-06 | 2.170E-43 | 5.426E-43 |
| 194.03 | 0.116 | 0.097 | 0.067 | 0.025 | 1.180E-05 | 8.157E-26 | 1.020E-25 |
| 388.24 | 0.106 | 0.082 | 0.065 | 0.003 | 1.019E-05 | 1.094E-42 | 1.824E-42 |
| 445.26 | 0.099 | 0.049 | 0.065 | 0.023 | 7.057E-06 | 3.268E-25 | 3.631E-25 |

Table S22. Performance of Classifiers for Discriminating LCa from HC (Discovery Set, Feature Panel).

| **Models** | **AUC** | **Accuracy** | **F1** | **Precision** | **Recall** |
| --- | --- | --- | --- | --- | --- |
| kNN | 0.936 | 0.869 | 0.869 | 0.869 | 0.869 |
| SVM | 0.960 | 0.885 | 0.884 | 0.901 | 0.885 |
| NN | 0.982 | 0.937 | 0.937 | 0.937 | 0.937 |
| NB | 0.940 | 0.859 | 0.859 | 0.863 | 0.859 |

Table S23. Performance of Classifiers for Discriminating LCa from HC (Validation Set, Feature Panel).

| **Models** | **AUC** | **Accuracy** | **F1** | **Precision** | **Recall** |
| --- | --- | --- | --- | --- | --- |
| kNN | 0.964 | 0.878 | 0.877 | 0.879 | 0.878 |
| SVM | 0.983 | 0.928 | 0.928 | 0.932 | 0.928 |
| NN | 0.981 | 0.937 | 0.936 | 0.939 | 0.937 |
| NB | 0.982 | 0.924 | 0.924 | 0.926 | 0.924 |

Table S24. Performance of the NN Classifier for LCa Staging (Discovery Set, Full Spectrum).

| **Models** | **AUC** | **Accuracy** | **F1** | **Precision** | **Recall** |
| --- | --- | --- | --- | --- | --- |
| kNN | 0.882 | 0.584 | 0.585 | 0.598 | 0.584 |
| SVM | 0.948 | 0.742 | 0.735 | 0.748 | 0.742 |
| NN | 0.964 | 0.832 | 0.831 | 0.832 | 0.832 |
| NB | 0.863 | 0.613 | 0.583 | 0.592 | 0.613 |

Table S25. Performance of the NN Classifier for LCa Staging (Validation Set, Full Spectrum).

| **Models** | **AUC** | **Accuracy** | **F1** | **Precision** | **Recall** |
| --- | --- | --- | --- | --- | --- |
| kNN | 0.938 | 0.699 | 0.701 | 0.715 | 0.699 |
| SVM | 0.964 | 0.839 | 0.835 | 0.849 | 0.839 |
| NN | 0.978 | 0.839 | 0.838 | 0.846 | 0.839 |
| NB | 0.895 | 0.602 | 0.569 | 0.605 | 0.602 |

Table S26. The 23 Key m/z Features Selected for Five-Group Respiratory Disease Classification. (* indicates metabolites putatively identified).

| **m/z feature** | **Info. gain** | **Gain ratio** | **Gini** | **ReliefF** | **FCBF** | **p-value** | **p-value (FDR adj)** |
| --- | --- | --- | --- | --- | --- | --- | --- |
| 365.11 | 0.388 | 0.217 | 0.097 | 0.030 | 2.365E-01 | 1.409E-173 | 3.240E-172 |
| 233.08 | 0.365 | 0.252 | 0.089 | 0.050 | 2.447E-01 | 9.200E-167 | 1.058E-165 |
| 231.05 | 0.319 | 0.250 | 0.087 | 0.043 | 2.197E-05 | 6.470E-144 | 4.960E-143 |
| 303.14 | 0.228 | 0.132 | 0.059 | 0.019 | 1.292E-01 | 2.997E-73 | 6.267E-73 |
| 388.24 | 0.206 | 0.144 | 0.049 | 0.021 | 1.256E-01 | 2.354E-106 | 1.083E-105 |
| 325.18* | 0.195 | 0.123 | 0.056 | 0.010 | 1.129E-05 | 2.736E-54 | 4.495E-54 |
| 332.25 | 0.183 | 0.129 | 0.047 | 0.003 | 1.105E-05 | 7.543E-39 | 8.674E-39 |
| 203.05 | 0.177 | 0.089 | 0.061 | 0.015 | 9.055E-06 | 5.354E-119 | 3.078E-118 |
| 379.06* | 0.159 | 0.082 | 0.036 | 0.018 | 8.180E-06 | 4.817E-38 | 5.275E-38 |
| 301.14 | 0.156 | 0.079 | 0.049 | 0.020 | 7.934E-06 | 1.184E-84 | 3.404E-84 |
| 194.03* | 0.145 | 0.136 | 0.038 | 0.013 | 9.510E-02 | 8.155E-105 | 3.126E-104 |
| 173.06 | 0.139 | 0.097 | 0.039 | 0.007 | 8.140E-06 | 6.234E-82 | 1.593E-81 |
| 221.04* | 0.139 | 0.072 | 0.035 | 0.016 | 7.070E-02 | 3.861E-77 | 8.880E-77 |
| 136.05* | 0.125 | 0.062 | 0.040 | 0.000 | 6.220E-06 | 4.468E-54 | 6.850E-54 |
| 184.07* | 0.125 | 0.073 | 0.036 | 0.015 | 6.694E-06 | 1.230E-48 | 1.489E-48 |
| 209.99 | 0.108 | 0.103 | 0.027 | 0.009 | 6.984E-06 | 1.182E-59 | 2.092E-59 |
| 368.21 | 0.108 | 0.193 | 0.030 | 0.002 | 8.260E-06 | 9.869E-52 | 1.335E-51 |
| 453.21 | 0.106 | 0.053 | 0.030 | 0.026 | 5.222E-06 | 3.050E-91 | 1.002E-90 |
| 217.10 | 0.104 | 0.131 | 0.030 | 0.007 | 7.286E-06 | 3.439E-69 | 6.591E-69 |
| 104.11* | 0.097 | 0.049 | 0.026 | 0.003 | 4.780E-06 | 3.545E-51 | 4.529E-51 |
| 445.26 | 0.084 | 0.042 | 0.026 | 0.006 | 4.106E-06 | 8.885E-54 | 1.277E-53 |
| 313.13* | 0.080 | 0.064 | 0.023 | 0.002 | 4.752E-02 | 1.193E-33 | 1.247E-33 |
| 269.14* | 0.024 | 0.021 | 0.006 | 0.016 | 1.432E-06 | 1.377E-08 | 1.377E-08 |

Table S27. Putative Identification of Nine Serum Metabolites by 15T FT-ICR MS.

| **Compound** | **Formula** | **Ions** | **Adduct m/z (FT ICR)** | **Theoretical adduct m/z** | **Delta** | **ppm** |
| --- | --- | --- | --- | --- | --- | --- |
| Choline | C_5_H_14_NO | [M]^+^ | 104.10734 | 104.10754 | -1.91E-06 | -1.91 |
| Histidine | C_6_H_9_N_3_O_2_ | [M+K]^+^ | 194.03263 | 194.03264 | -2.58E-08 | -0.03 |
| Hydroxydodecanedioic acid | C_12_H_22_O_5_ | [M+Na]^+^ | 269.13593 | 269.13594 | -4.46E-08 | -0.04 |
| Lysylglutamine | C_11_H_22_N_4_O_4_ | [M+K]^+^ | 313.12720 | 313.12726 | -2.01E-07 | -0.20 |
| Trimethylammoniobutanoic acid | C_7_H_15_NO_2_ | [M+K]^+^ | 184.07332 | 184.07344 | -6.36E-07 | -0.64 |
| Dihydroxycinnamic acid glucuronide | C_15_H_16_O_10_ | [M+Na]^+^ | 379.06410 | 379.06357 | 1.41E-06 | 1.41 |
| Hexadecanedioic acid | C_16_H_30_O_4_ | [M+K]^+^ | 325.17758 | 325.17757 | 4.00E-08 | 0.04 |
| Vanillylmandelic acid | C_9_H_10_O_5_ | [M+Na]^+^ | 221.04221 | 221.04204 | 7.65E-07 | 0.76 |
| Creatinine | C_4_H_7_N_3_O | [M+Na]^+^ | 136.04816 | 136.04813 | 2.21E-07 | 0.22 |

Table S28. Performance of Classifiers for Five-Group Classification (Discovery Set, Full Spectrum).

| **Models** | **AUC** | **Accuracy** | **F1** | **Precision** | **Recall** |
| --- | --- | --- | --- | --- | --- |
| kNN | 0.950 | 0.819 | 0.814 | 0.819 | 0.819 |
| SVM | 0.973 | 0.840 | 0.840 | 0.850 | 0.840 |
| NN | 0.978 | 0.909 | 0.909 | 0.909 | 0.909 |
| NB | 0.924 | 0.728 | 0.726 | 0.727 | 0.728 |

Table S29. Performance of Classifiers for Five-Group Classification (Validation Set, Full Spectrum).

| **Models** | **AUC** | **Accuracy** | **F1** | **Precision** | **Recall** |
| --- | --- | --- | --- | --- | --- |
| kNN | 0.958 | 0.850 | 0.847 | 0.852 | 0.850 |
| SVM | 0.960 | 0.839 | 0.838 | 0.864 | 0.839 |
| NN | 0.990 | 0.923 | 0.924 | 0.925 | 0.923 |
| NB | 0.918 | 0.631 | 0.643 | 0.741 | 0.631 |

Table S30. Performance of Classifiers for Five-Group Classification (Discovery Set, 23-Metabolite Panel).

| **Models** | **AUC** | **Accuracy** | **F1** | **Precision** | **Recall** |
| --- | --- | --- | --- | --- | --- |
| kNN | 0.886 | 0.698 | 0.697 | 0.698 | 0.698 |
| SVM | 0.925 | 0.739 | 0.736 | 0.754 | 0.739 |
| NN | 0.950 | 0.806 | 0.805 | 0.805 | 0.806 |
| NB | 0.908 | 0.698 | 0.698 | 0.706 | 0.698 |

Table S31. Performance of Classifiers for Five-Group Classification (Validation Set, 23-Metabolite Panel).

| **Models** | **AUC** | **Accuracy** | **F1** | **Precision** | **Recall** |
| --- | --- | --- | --- | --- | --- |
| kNN | 0.905 | 0.720 | 0.716 | 0.719 | 0.720 |
| SVM | 0.918 | 0.738 | 0.731 | 0.752 | 0.738 |
| NN | 0.956 | 0.817 | 0.814 | 0.813 | 0.817 |
| NB | 0.910 | 0.712 | 0.707 | 0.711 | 0.712 |
